# *Mycobacterium marinum* antagonistically induces an autophagic response while repressing the autophagic flux in a TORC1- and ESX-1-dependent manner

**DOI:** 10.1371/journal.ppat.1006344

**Published:** 2017-04-17

**Authors:** Elena Cardenal-Muñoz, Sonia Arafah, Ana Teresa López-Jiménez, Sébastien Kicka, Alexandra Falaise, Frauke Bach, Olivier Schaad, Jason S. King, Monica Hagedorn, Thierry Soldati

**Affiliations:** 1 Department of Biochemistry, Faculty of Sciences, Sciences II, University of Geneva, Geneva, Switzerland; 2 Section Parasitology, Bernhard Nocht Institute for Tropical Medicine, Hamburg, Germany; 3 Centre for Membrane Interactions and Dynamics, Department of Biomedical Sciences, University of Sheffield, Firth Court, Western Bank, Sheffield, United Kingdom; 4 Bateson Centre, University of Sheffield, Firth Court, Western Bank, Sheffield, United Kingdom; Portland VA Medical Center, Oregon Health and Science University, UNITED STATES

## Abstract

Autophagy is a eukaryotic catabolic process also participating in cell-autonomous defence. Infected host cells generate double-membrane autophagosomes that mature in autolysosomes to engulf, kill and digest cytoplasmic pathogens. However, several bacteria subvert autophagy and benefit from its machinery and functions. Monitoring infection stages by genetics, pharmacology and microscopy, we demonstrate that the ESX-1 secretion system of *Mycobacterium marinum*, a close relative to *M*. *tuberculosis*, upregulates the transcription of autophagy genes, and stimulates autophagosome formation and recruitment to the mycobacteria-containing vacuole (MCV) in the host model organism *Dictyostelium*. Antagonistically, ESX-1 is also essential to block the autophagic flux and deplete the MCV of proteolytic activity. Activators of the TORC1 complex localize to the MCV in an ESX-1-dependent manner, suggesting an important role in the manipulation of autophagy by mycobacteria. Our findings suggest that the infection by *M*. *marinum* activates an autophagic response that is simultaneously repressed and exploited by the bacterium to support its survival inside the MCV.

## Author summary

One of the cell-autonomous defence pathways against intracellular pathogens is autophagy, an ancestral eukaryotic process surprisingly conserved throughout evolution. Recent studies have highlighted contradictory roles for autophagy during mycobacterial infection. Whereas some studies revealed a role for autophagy to control intracellular bacterial growth, others brought evidence that mycobacteria somehow inhibit autophagic killing. Here, we demonstrate for the first time that *Mycobacterium marinum* induces both an early autophagic response and its simultaneous repression by blocking the autophagic digestion. This antagonistic manipulation of autophagy is dependent on a functional ESX-1-secretion system, which secretes the membrane-damaging factor ESAT-6, proposed to participate in the perforation of the *M*. *marinum-*containing vacuole (MCV). We show here that these membrane damages activate the formation of autophagosomes and their recruitment to the MCV. However, *M*. *marinum* also utilizes its ESX-1 secretion system to avoid killing inside autolysosomes by blocking the autophagic flux. In addition, we bring evidence that this manipulation of autophagy is orchestrated via the regulation of TORC1, the major eukaryotic kinase complex controlling nutrient-sensing and cell metabolism.

## Introduction

Autophagy is one of the most ancestral and important catabolic pathways in eukaryotes. Under stresses such as oxidative stress, nutrient starvation, accumulation of toxic protein aggregates or DNA and organelles damage, cells turn on a complex machinery to maintain their homeostasis. One of the main protein complexes regulating autophagy is TORC1 (target of rapamycin complex 1), which comprises the serine/threonine kinase TOR that coordinates cell growth and metabolism [1]. Under optimal conditions, TORC1 suppresses autophagy, while promoting growth via increased ribosome biogenesis and protein translation. However, upon stress, TORC1 activity is repressed and autophagy generates nutrients and energy to maintain essential activities [2]. When TORC1 is inhibited, the Atg1/ULK1 kinase complex activates and promotes the recruitment of Atg8/LC3 and Atg18/WIPI-2 to autophagosome formation sites (next to the vacuole in yeasts, and at multiple locations in mammalian cells and the amoeba *Dictyostelium discoideum*) [3]. Atg8 is lipidated and incorporated into both the external and internal sides of the phagophore membrane, facilitating its expansion and engulfment of cytoplasmic material into a double-membrane vesicle termed autophagosome. After fusion between autophagosomes and lysosomes, the sequestered material is digested and recycled into new macromolecules [4].

As a degradation pathway, autophagy can also operate as an innate immune response against intracellular pathogens, specifically coined "xenophagy". The pathogen or the damaged cellular components activate autophagy, causing the capture and digestion of the invader [5]. Nevertheless, some microbes exploit the autophagic machinery to their own benefit, such as *Francisella tularensis* or *Staphylococcus aureus*, which use autophagy to support their intracellular growth [6, 7]. *Mycobacterium tuberculosis*, a major threat to human health and causative agent of tuberculosis, survives in host cells by arresting phagosome maturation [8]. *M*. *tuberculosis* can escape into the host cytosol by damaging the membrane of its containing compartment [9, 10]. Autophagy, shown to be induced by *M*. *tuberculosis* infection, controls mycobacterial growth in host cells [11, 12]. However, these bacteria also harbour several lipidic and proteinic virulence factors such as lipoarabinomannan (LAM), PDIMs, Eis, Rv3242c, Rv3167c, SapM, PhoP and the type VII secretion system ESX-1, to inhibit killing by autophagy [13–19].

The role of the ESX-1 secretion system in the regulation of autophagy has also been studied during *Mycobacterium marinum* infection. *M*. *marinum* is a close relative of *M*. *tuberculosis* that naturally infects fish and frogs and produces skin lesions in humans [20]. Its conserved ESX-1 secretion system is essential for its escape from the mycobacteria-containing vacuole (MCV) and induction of an autophagic response in the host [21, 22]. However, whether *M*. *marinum* ESX-1 is also involved in the inhibition of the autophagic flux, as described for *M*. *tuberculosis*, remains unexplored. Here, by using *D*. *discoideum*/*M*. *marinum* as a model system to study mycobacterial infections, we demonstrate for the first time that *M*. *marinum* induces both an early autophagic response and its simultaneous repression by blocking the autophagic flux. This antagonistic manipulation of autophagy is dependent on its ESX-1-secretion system.

Free-living *D*. *discoideum* is a biochemically and genetically tractable amoeba that phagocytoses bacteria and yeasts for food. It possesses a simplified and well-conserved cell-intrinsic immune system, making this professional phagocyte a powerful model to study host cell responses during bacterial infection [23]. Most mammalian autophagy genes are conserved in *D*. *discoideum*, permitting the use of this amoeba to uncover the mechanisms of xenophagy against a variety of pathogens [24]. For instance, we have shown that after escape to the cytosol *M*. *marinum* recruits the autophagic machinery to egress from *D*. *discoideum* in non-lytic manner [25]. Here, we reveal the kinetics of the *D*. *discoideum* xenophagic response to *M*. *marinum* and show that these virulent mycobacteria induce autophagy at the level of gene transcription, autophagosome formation and recruitment to the MCV, while blocking the autophagic flux in an ESX-1- and TORC1-dependent manner.

## Results

### *M*. *marinum* induces an early autophagic response in *D*. *discoideum*

**Fig 1:**
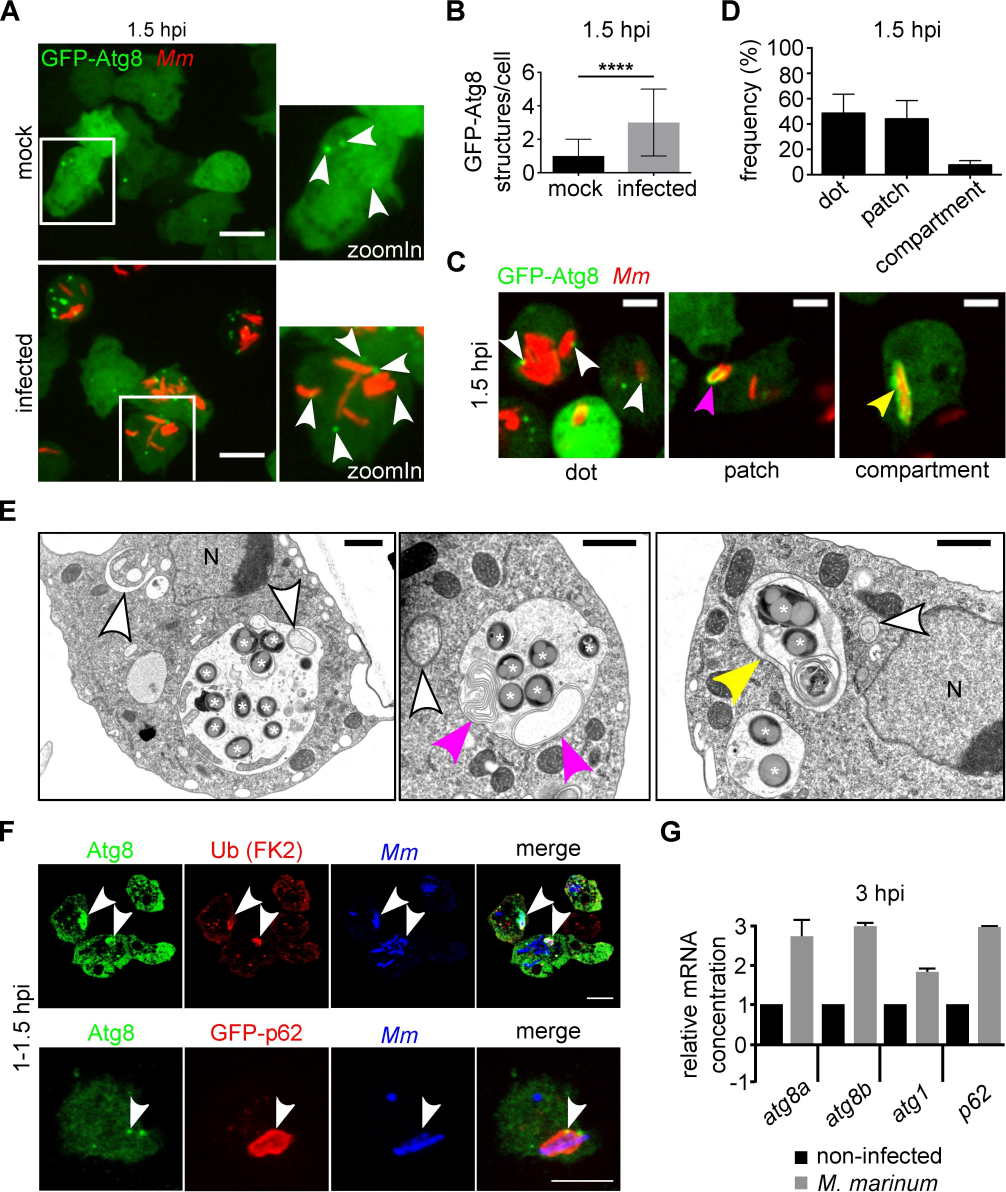
*M*. *marinum* induces an early autophagic response in *D*. *discoideum*. **A.** Two representative maximum projections showing GFP-Atg8-expressing cells infected (bottom) or mock-infected (top) with mCherry-expressing wt *M*. *marinum*. Images were recorded live 1.5 h after infection. White arrowheads point to GFP-Atg8 structures. Scale bars, 10 μm; **B.** The median and interquartile ranges of the number of GFP-Atg8 structures per cell were calculated. For each condition, 300 cells from independent triplicates were counted. Mann-Whitney test (****p ≤ 0.0001); **C.** Single sections of live GFP-Atg8-expressing amoebae 1.5 h after infection with mCherry-expressing *M*. *marinum* wt. White, magenta and yellow arrowheads point to GFP-Atg8 dots, patches and GFP-Atg8-positive (GFP-Atg8^+^) MCV, respectively. Scale bars, 5 μm; **D.** At 1.5 hpi, 256 cells (100%) with GFP-Atg8^+^ *M*. *marinum* were classified as in **C**. Means and standard deviations from three independent infections are represented; **E.** EM of the different types of autophagosomes in the vicinity of the MCV at 7 hpi. White asterisks label bacteria, white arrowheads point to round phagophores and autophagosomes, magenta arrowheads point to extended autophagosomes, and the yellow arrowhead indicates a double membrane compartment containing mycobacteria. Nuclei <N>. Scale bars, 1 μm; **F.** Sections (top) and maximum projections (bottom) showing co-localization (white arrowheads) of autophagy markers with bacteria at 1–1.5 hpi. Around 50% and 30% of the Atg8-positive bacteria were also positive for Ub and GFP-p62, respectively. Scale bars, 5 μm; **G.** qPCR results of relative abundance of *atg8a*, *atg8b*, *atg1* and *p62* mRNAs at 3 hpi. The mRNA level in non-infected cells is indicated as 1. The mRNA level of the housekeeping gene *gapdh* was used for normalization. Means and standard deviations from three independent experiments performed in triplicates are represented.

Autophagy is highly dynamic in *D*. *discoideum*, as evidenced by the rapid flow of GFP-Atg8^+^ autophagosomes from formation to degradation, and the fusion events, which occur in 1–3 min (S1A–S1C Fig). Thus, the average number of autophagosomes at steady state can be as low as one (Fig 1A and 1B, mock). Under autophagy-inducing conditions, such as nutrient starvation [26], mechanical stress [27] and rapamycin treatment [28], Atg8 associates with nascent autophagosomes, resulting in an increase in the number of GFP-Atg8 puncta. To investigate whether *M*. *marinum* induced a similar response, we infected GFP-Atg8-expressing *D*. *discoideum* cells with wild type (wt) bacteria. By live microscopy, we detected a three-fold increase in the number of GFP-Atg8 structures 1.5 hours post-infection (hpi) (Fig 1A and 1B). This three-fold increase was confirmed using GFP-Atg18 (S1D–S1E Fig), a marker specific for omegasomes and expanding phagophores [29].

Notably, recruitment of GFP-Atg8 to the bacteria fell in distinct patterns that we defined as dots (puncta next to the bacterium), patches (extended structures along the bacterium) and compartments (vacuoles completely surrounding the bacterium) (Fig 1C and 1D). Double-membrane structures were observed by EM of the MCV (Fig 1E), likely corresponding to those detected by fluorescence microscopy (Fig 1C). This heterogeneity in GFP-Atg8^+^ patterns next to the MCV might reflect temporal stages in a rapid series of events during phagophore elongation around the MCV and/or MCV-autophagosome fusion. In addition, immunofluorescence staining showed co-localization of the autophagy markers ubiquitin (Ub) and p62 with Atg8 at the site of recruitment to bacteria (Fig 1F), suggesting that the classic autophagic machinery was activated early after infection.

The accumulation of autophagosomes during infection might be indicative of either increased induction or decreased degradation, since an impairment of autolysosomal function would also lead to the accumulation of autophagy proteins and compartments [30]. A change in the mRNA levels of autophagy genes may correlate with the change in autophagic activity [30]. Therefore, we measured the transcription levels of autophagy genes at various stages of infection. The expression of the two *D*. *discoideum atg8* genes (*atg8a* and *atg8b)*, of *atg1* and *p62* was significantly upregulated early after infection (Fig 1G).

### Autophagy induction restricts *M*. *marinum* proliferation

**Fig 2:**
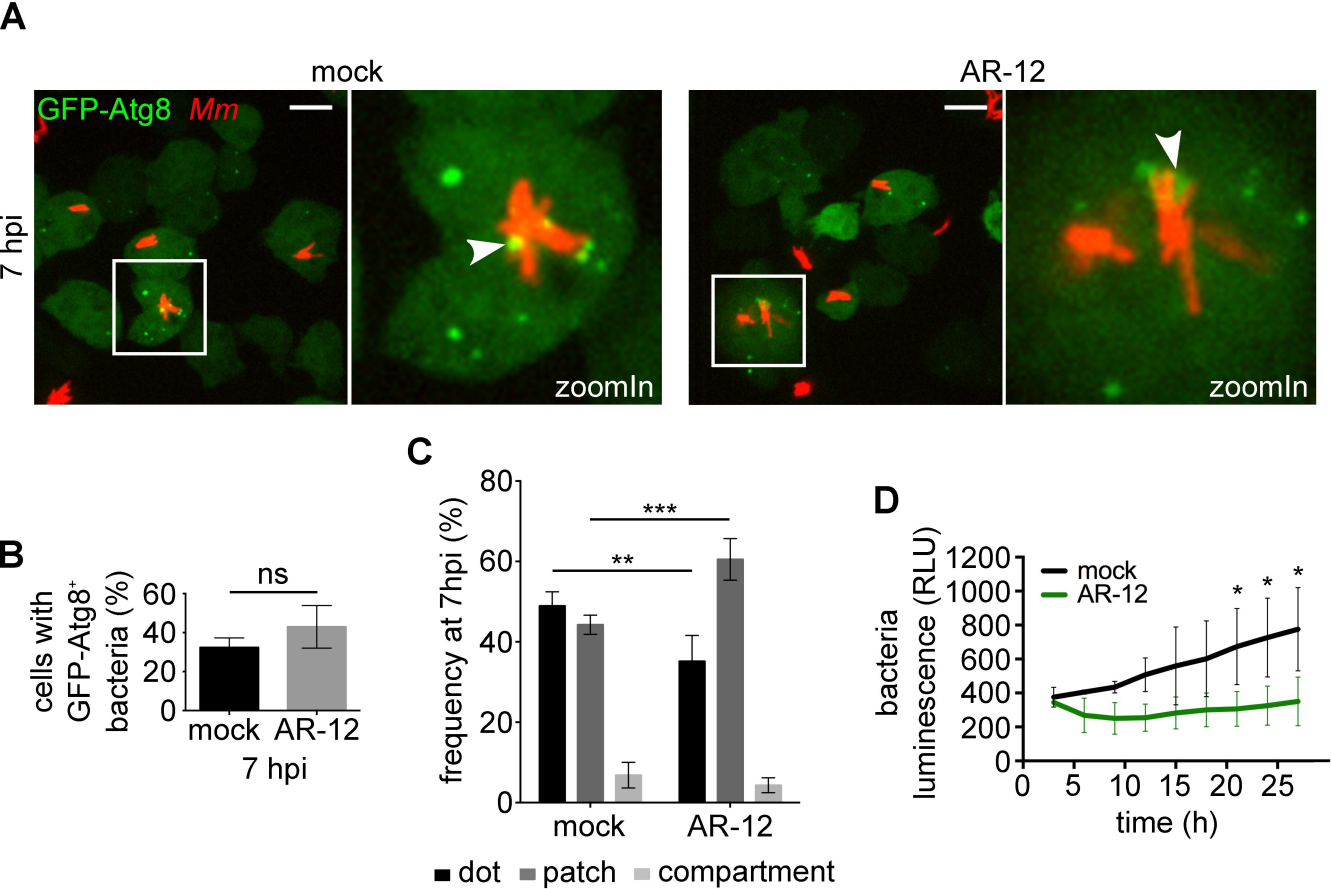
Artificial induction of autophagy restricts *M*. *marinum* proliferation. **A.** GFP-Atg8-expressing cells were infected for 5 h with mCherry-expressing *M*. *marinum* wt and treated or not with AR-12 at 2.5 μM for 2 additional hours. Representative maximum projections of live imaging are shown. White arrowheads point to GFP-Atg8 recruitment to MCV. Scale bars, 10 μm; **B.** Quantification of the percentage of infected cells with GFP-Atg8^+^ bacteria at 7 hpi. Means and standard deviations from six (mock) and three (AR-12) independent replicates are represented and an unpaired *t* test showed no statistical significance (ns, *p* > 0.05). 688 and 297 infected cells were counted for the mock and the AR-12 treatments, respectively; **C.** Classification of types of GFP-recruitment for infected mock (258 cells) and AR-12 (141 cells) treated. Means and standard deviations from six (mock) and three (AR-12) independent replicates are represented. Unpaired *t* test (**p ≤ 0.01; ***p ≤ 0.001); **D.** Cells infected with *lux*-expressing *M*. *marinum* wt bacteria were treated or not with AR-12 at 2.5 μM. Intracellular bacteria growth [relative luminescence units (RLU)] is represented as the mean and standard deviation from triplicates. Statistical differences were calculated with a Bonferroni post hoc test after two-way ANOVA (*p ≤ 0.05).

To determine the impact of autophagy induction during infection, we treated infected cells with the autophagy inducer drug AR-12/OSU-03012 [31]. After validating that AR-12 induced the formation of autophagosomes (S2A–S2C and S6D–S6E Figs), and was neither cytotoxic for *D*. *discoideum* nor for *M*. *marinum* (S2D–S2E Fig), we quantified the recruitment of GFP-Atg8 to *M*. *marinum* after drug treatment (Fig 2A–2C). We did not observe a significant difference in its overall recruitment (Fig 2B), but the number of patches increased by 20% when compared to dots, suggesting enhanced phagophore elongation around the bacteria (Fig 2C). Formation of GFP-Atg8 vacuoles around the bacteria did not increase though, suggesting that the xenophagic flow was somehow arrested. In addition, treatment with AR-12 lead to a decrease in bacterial load, as measured using *lux*-expressing *M*. *marinum* (Fig 2D), suggesting that artificial induction of autophagy can restrict *M*. *marinum* survival and/or proliferation. The decrease in bacterial load resulting from artificial induction of autophagy was confirmed with other inducers such as the TOR kinase inhibitors AZD8055 and PI-103 [32, 33] (S2F Fig).

### The autophagic machinery is required to maintain the MCV

**Fig 3:**
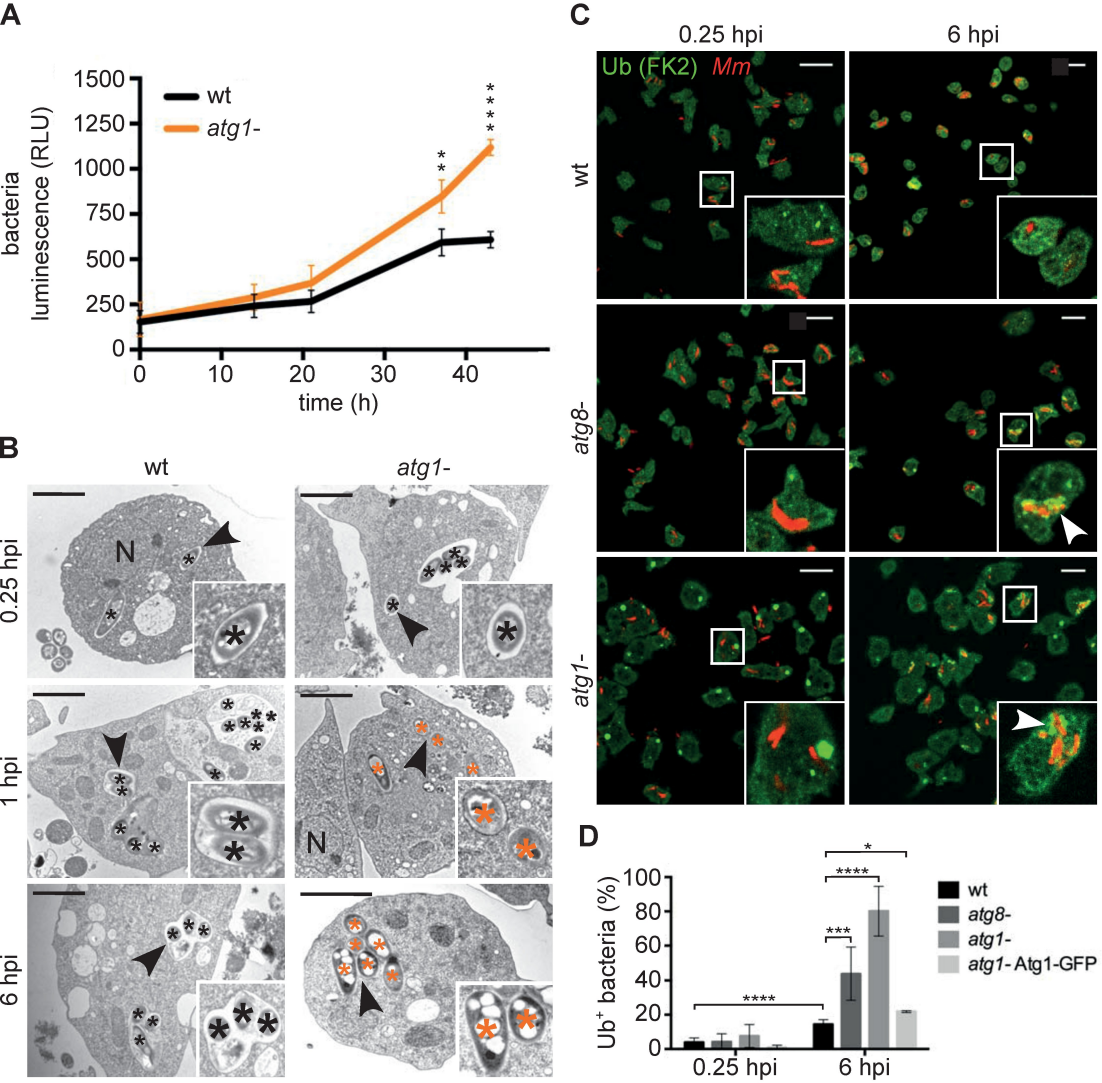
Autophagy is necessary for the maintenance of the MCV. **A.** The intracellular growth of *lux*-expressing *M*. *marinum* wt was monitored inside wt and *atg1*- cells. RLUs are represented as the mean and standard deviation from quadruplicates. Statistical differences were calculated with a Bonferroni post hoc test after two-way ANOVA (**p ≤ 0.01; (****p ≤ 0.0001); **B.** EM of the locations of *M*. *marinum* wt inside wt and *atg1*- cells at 0.25, 1 and 6 hpi. Black and orange asterisks label bacteria inside a compartment or in the cytosol, respectively. Black arrowheads mark the zoomIn. Nuclei <N>. Scale bars, 2 μm; **C.** *D*. *discoideum* wt, *atg8*- and *atg1*- cells were infected with mCherry-expressing *M*. *marinum*, fixed and stained against Ub (green) and mCherry (red). Representative maximum projections at 0.25 and 6 hpi are shown. White arrowheads label the ubiquitinated bacteria. Scale bars, 10 μm; **D.** Quantification of the percentage of intracellular bacteria/MCVs (red) positive for Ub (green) in wt, *atg8*-, *atg1*- and *atg1*-Atg1-GFP cells at 0.25 and 6 hpi. Means and standard deviations from 2–3 independent experiments. 128–299 infected cells were counted per time point and cell line. Unpaired *t* test (*p ≤ 0.05; ***p ≤ 0.001; **** *p* ≤ 0.0001).

In *D*. *discoideum*, disruption of *atg1*, the homolog of the mammalian ULK1 kinase, leads to a complete block in the canonical pathway [34]. To confirm a negative effect of autophagy on the proliferation of *M*. *marinum*, we infected wt and *atg1*- cells with *lux*-expressing bacteria. As predicted, the bacterial load was significantly higher in the *atg1* mutants, visible from 24 hpi (Fig 3A). This increase was also observed during infection of *atg8* knock out cells, but not of cells lacking the selective autophagy adaptor p62 (S3A Fig), because of potential redundancy with other Ub-binding xenophagy adaptors [24]. Importantly, treatment with AR-12 had no effect on bacterial load in *atg1*- cells, confirming the autophagy-dependent effect of this drug (S3B Fig).

We have previously shown that *M*. *marinum* grows inside a vacuole with features of post-lysosomes in *D*. *discoideum*, and escapes into the cytosol after about 24 hpi [10, 35]. In addition, we show here that autophagic structures accumulate around bacteria (Fig 1C). We therefore wondered whether autophagy provides membranes to maintain the integrity of the MCV, as previously suggested for other bacteria [36, 37]. Surprisingly, EM monitoring of infected *atg1*- cells revealed that *M*. *marinum* was released into the cytosol as early as 1 hpi (Fig 3B). Cytosolic, but not vacuolar *M*. *marinum* becomes ubiquitinated in both macrophages and *D*. *discoideum* [25, 38]. Staining for p80, an endosomal copper transporter enriched at the MCV [35], also distinguishes between bacteria inside a phagosomal compartment (p80-positive and non-ubiquitinated), and cytosolic bacteria (p80-negative and positive for Ub) (S4A Fig). Consistent with our EM data, we detected a striking increase in the level of ubiquitinated *M*. *marinum* in *atg8*- and *atg1*- cells (Fig 3C and 3D), which was complemented by expression of Atg1-GFP (Figs S4B and 3D). Taken together, these results suggest that autophagy is necessary for the establishment and/or maintenance/repair of the MCV, preventing early escape of mycobacteria to the cytosol.

### *M*. *marinum* induces autophagosome formation in an ESX-1-dependent manner

**Fig 4:**
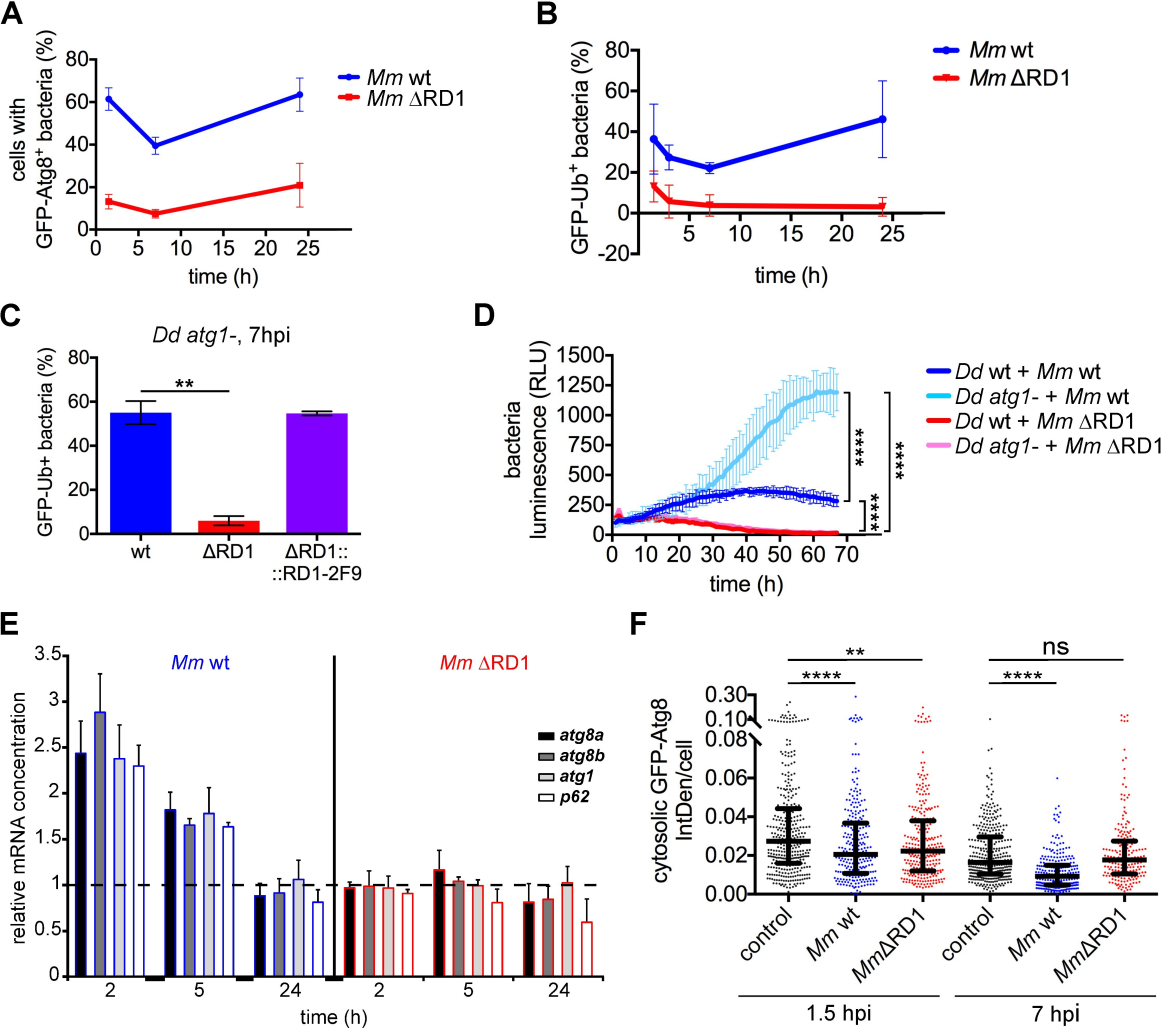
The early autophagic response caused by *M*. *marinum* infection is dependent on ESX-1. **A.** Percentage of cells containing GFP-Atg8^+^ *M*. *marinum* wt and ΔRD1 at 1.5, 7 and 24 hpi. Means and standard deviations from independent triplicates. A mean of 375 and 230 infected cells per time point was counted for *M*. *marinum* wt and ΔRD1 infection, respectively; **B.** Percentage of *M*. *marinum* wt and ΔRD1 bacteria positive for GFP-Ub. Mean and standard deviation from independent triplicates; **C.** GFP-Ub-expressing *atg1*- cells were infected with Vibrant DyeCycle Ruby-labelled *M*. *marinum* wt, ΔRD1 and ΔRD1::2F9. Means and standard deviations from independent duplicates of the percentage of bacteria positive for GFP-Ub at 7 hpi. A mean of 72–98 bacteria per infection was counted. Unpaired *t* test (**p ≤ 0.01); **D.** *D*. *discoideum* wt and *atg1*- cells were infected with *lux*-expressing *M*. *marinum* wt and ΔRD1. The intracellular bacteria growth (RLUs) from triplicates was monitored. Statistical differences at the end of the experiment were calculated with a Bonferroni post hoc test after two-way ANOVA (****p ≤ 0.0001); **E.** qPCR results of relative abundance of *atg8a*, *atg8b*, *atg1* and *p62* mRNAs at 2, 5 and 24 hpi with *M*. *marinum* wt (blue outline) and ΔRD1 (red outline). The mRNA level in non-infected cells is indicated as 1 in the figure (dashed line). The mRNA level of the housekeeping gene *gapdh* was used for normalization. Means and standard deviations from three independent experiments performed in triplicates are represented; **F.** GFP-Atg8-expressing cells were infected or mock-infected for 1.5 or 7 h with mCherry-expressing *M*. *marinum* wt or with DsRed-expressing *M*. *marinum* ΔRD1. Maximum projections were used to measure the Integrated Density (IntDen) of the cytosolic GFP-Atg8 fluorescence compared to the extracellular IntDen (background). Median with interquartile ranges of the cytosolic GFP-Atg8 IntDen per cell. Each dot represents one cell. 210–357 cells per condition from three independent experiments were counted. Mann-Whitney test (****p ≤ 0.0001; **p ≤ 0.01; ns, *p* > 0.05).

Both *M*. *tuberculosis* and *M*. *marinum* possess a genomic locus, RD1/ESX-1, that encodes a type VII system necessary for secretion of the virulence factor ESAT-6, proposed to participate in the disruption of membranes in mammalian cells [9, 39] as well as in the rupture of the MCV in *D*. *discoideum* [10]. Because membrane damage caused by various bacteria induces autophagy [40], we hypothesize that ESAT-6 might also be responsible for the recruitment of the autophagic machinery to early MCVs. Monitoring GFP-Atg8 and GFP-Ub during the first stages of infection revealed a considerable decrease of their recruitment to the ΔRD1 bacteria, from 60% to 20% and from 40% to 20%, respectively (Figs 4A and 4B and S4C). Infection of *atg1*- cells with either *M*. *marinum* ΔRD1 or ΔCE, a strain with a knock out restricted to ESAT-6 and its chaperone CFP-10 [41], also showed reduced bacteria ubiquitination at 6–7 hpi (Figs 4C and S4D), suggesting that secretion of ESAT-6 is thus required for cytosolic escape and subsequent ubiquitination of mycobacteria in *D*. *discoideum*. Complementation of the ΔRD1 strain with the *M*. *tuberculosis*-derived RD1-2F9 cosmid [42] restored ubiquitination to wt levels (Fig 4C).

In addition, we measured intracellular proliferation of *lux*-expressing *M*. *marinum* wt and ΔRD1 strains (Fig 4D). Deletion of the RD1 locus severely reduced the *M*. *marinum* load in both wt and *atg1*- cells, suggesting that the growth advantage of wt bacteria in *atg1*- *D*. *discoideum* strictly depends on their capacity to escape to the cytosol. Moreover, the upregulation of autophagy genes early after infection (Fig 4E) was also strictly dependent on a functional ESX-1 secretion system. We also observed the reduction in fluorescence intensity of the cytosolic GFP-Atg8 fraction in cells infected with *M*. *marinum* wt but not with ΔRD1 (Fig 4F), indicating that the activity of the RD1 locus induces autophagosome formation and the consequent translocation of cytosolic GFP-Atg8 to membranes. Therefore, we suggest that the damages produced by ESAT-6 to the MCV membrane lead to an early induction of the autophagic response, which somehow limits damage and contributes to retain bacteria in the MCV.

### *M*. *marinum* blocks the autophagic flux in *D*. *discoideum*

**Fig 5:**
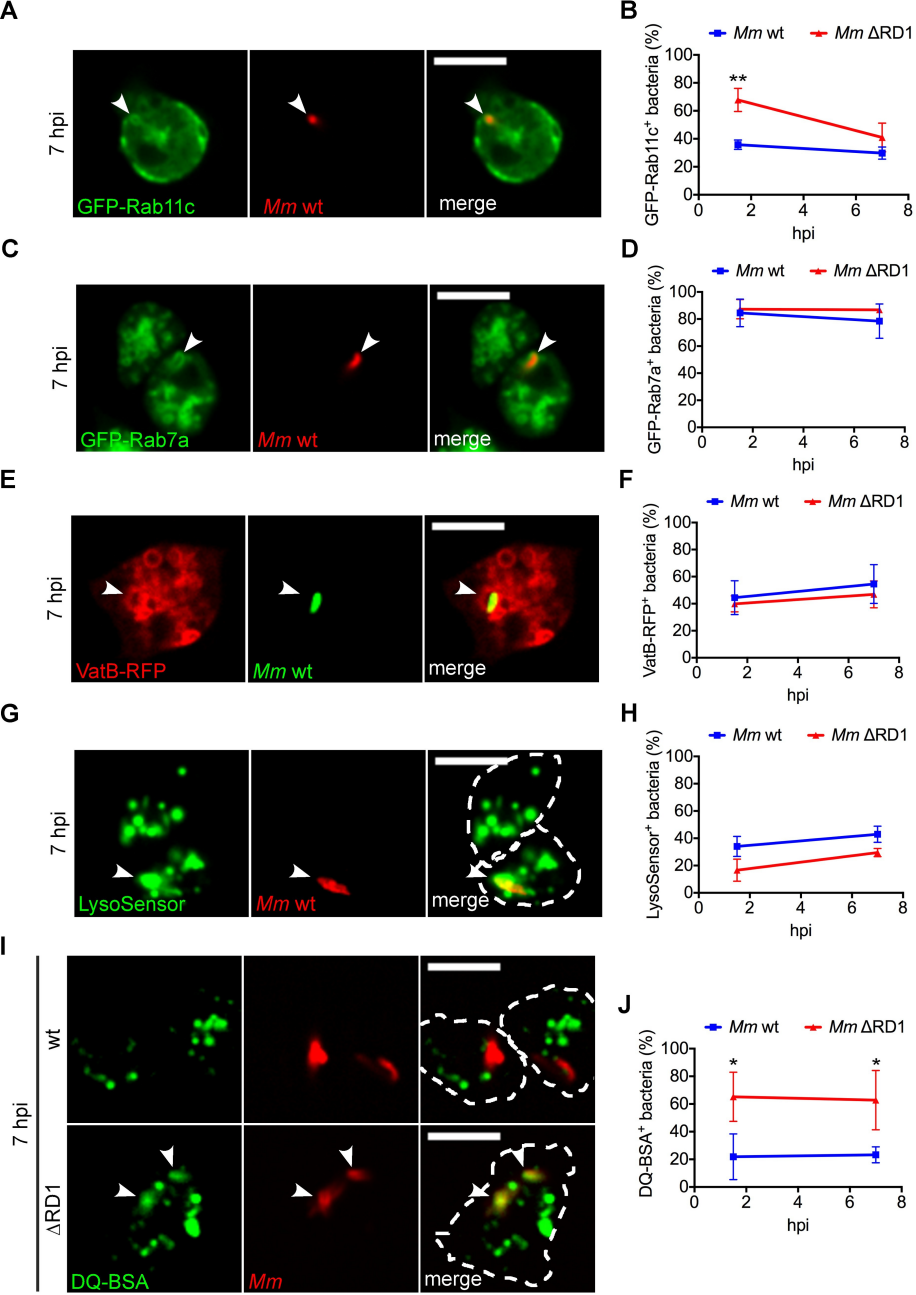
Expression of ESX-1 is essential to devoid the MCV of proteolytic activity. Cells expressing GFP-Rab11c, GFP-Rab7a or VatB-RFP, or incubated with LysoSensor Green or DQ Green BSA were infected for 1.5 and 7h with *M*. *marinum* wt or ΔRD1 labelled with Vibrant DyeCycle Ruby or expressing mCherry, DsRed or GFP. Representative sections of live imaging at 7 hpi are shown in **A**, **C**, **E**, **G** and **I**. White arrowheads point to the sites of recruitment/co-localization to the MCV. Scale bars, 10 μm; The percentage of bacteria/MCVs positive for GFP-Rab11c (**B**), GFP-Rab7a (**D**), VatB-RFP (**F**), LysoSensor Green (**H**) or DQ Green BSA (**J**) at 1.5 and 7 hpi was quantified. Mean and standard deviation from 2–3 independent experiments. A minimum of 100 infected cells were counted for each cell line. Unpaired *t* test compared wt and ΔRD1 bacteria (*p ≤ 0.05; **p ≤ 0.01).

Because *M*. *marinum* induces the formation of autophagosomes and xenophagy represses bacteria growth, the expected bacterial fate would be degradation in autolysosomes. However, intracellular mycobacteria not only survive but proliferate. One plausible explanation is that *M*. *marinum* avoids xenophagy by blocking the autophagic flux, as already suggested by the lack of effect of AR-12 on the frequency of GFP-Atg8^+^ compartments fully enclosing bacteria (Fig 2C). We first mapped the endocytic and autophagic features of the MCVs containing *M*. *marinum* (Fig 5). At 7 hpi, only 30% of the MCVs were decorated by Rab11, a recycling endosome GTPase involved in phagophore elongation and autophagosome maturation, while 80% of the compartments recruited Rab7, a late endosomal protein necessary for the transition from autophagosomes to autolysosomes [43] (Fig 5A–5D). In addition, 40–50% of the MCVs were positive for markers of acidic compartments such as VatB, a peripheral subunit of the vacuolar H^+^-ATPase, and LysoSensor Green (Fig 5E–5H). We did not observe any significant difference in these characteristics between the wt and the ΔRD1 MCVs, even though there is no overall proliferation of the latter mutant bacteria (Fig 4D). In sharp contrast, staining with DQ-BSA revealed that only the *M*. *marinum* ΔRD1 MCV had lysosomal-like degradative capacity (Fig 5I and 5J). Together, these data indicate that, at the time points studied here, *M*. *marinum* wt actually resides in a compartment with features of late endosomes, and suggest that a functional ESX-1 is necessary to block either fusion of the MCV with lysosomes or the activity of lysosomal enzymes.

**Fig 6:**
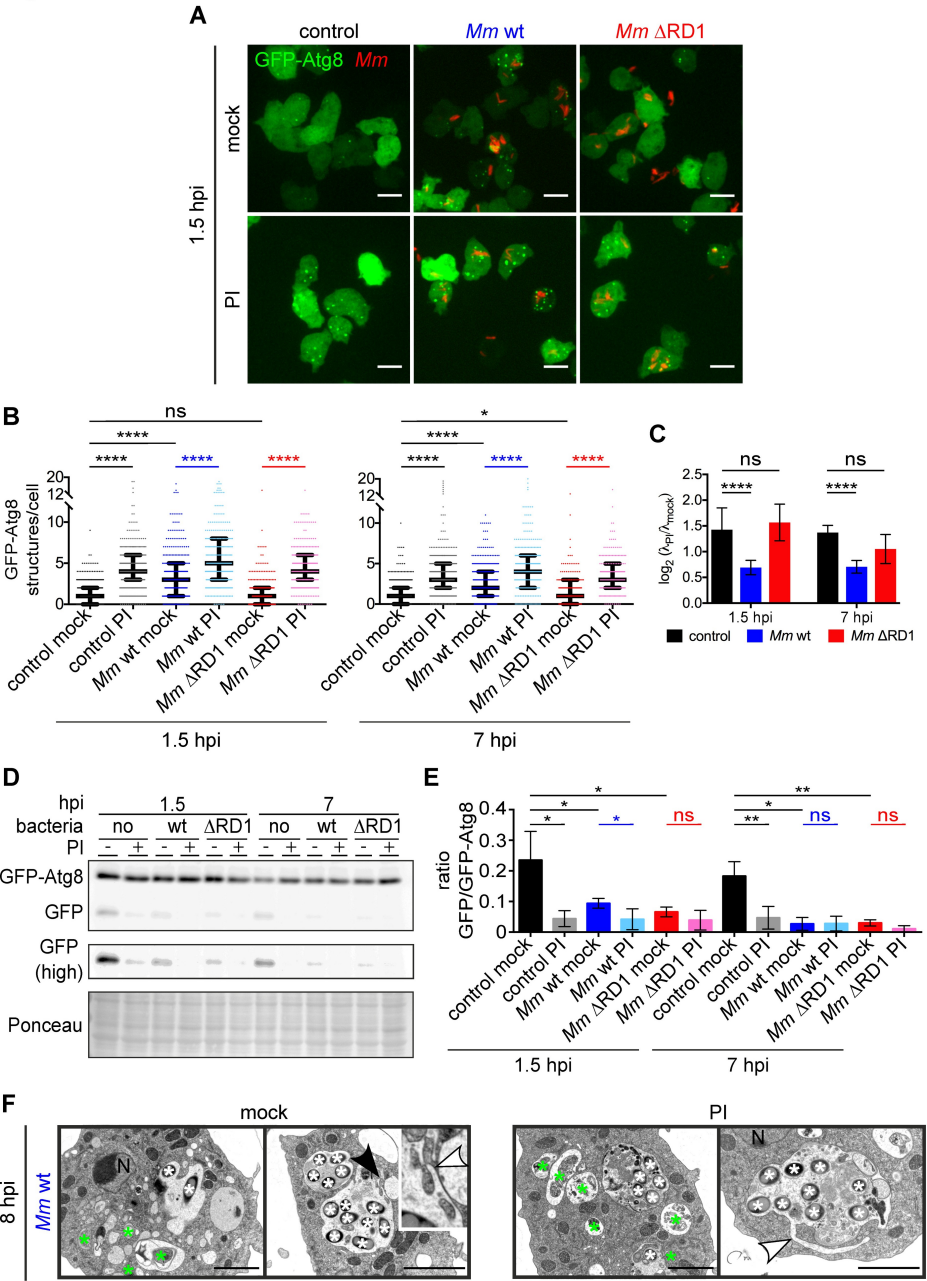
The autophagic flux is blocked during wt *M*. *marinum* infection. **A.** GFP-Atg8-expressing cells were infected or mock-infected for 0.5 or 6 h with mCherry-expressing *M*. *marinum* wt or with DsRed-expressing *M*. *marinum* ΔRD1 and treated or not with a PI cocktail at 2.5× for one additional hour. Representative maximum projections of live imaging at 1.5 hpi are shown. Scale bars, 10 μm; **B.** Medians with interquartile ranges of the number of GFP-Atg8 structures per cell from the infections carried out in **A**. Each dot represents one cell. 178–338 cells per condition from three independent experiments were counted. The λ that define the Poisson distribution of each data set and differences between them were calculated as described in Materials and Methods (*p ≤ 0.05; ****p ≤ 0.0001; ns, *p* > 0.05); **C.** Mean and standard deviation of the log_2_ (**λ**_PI_/**λ**_mock_) from the three independent replicates represented in **B**. A log_2_ (**λ**_PI_/**λ**_mock_) of zero implies that there was a total autophagic block. *p*-values calculated as described in Materials and Methods (****p ≤ 0.0001; ns, *p* > 0.05). **D.** Samples from infections (**A**) were immunoblotted against GFP. Longer exposure of the free GFP bands is shown as "GFP (high)". Ponceau-S staining as loading control. Representative result from four independent experiments **E.** Means and standard deviations of the ratio GFP/GFP-Atg8 from the immunoblots represented in **D**. Unpaired *t* test (*p ≤ 0.05; **p ≤ 0.01; ns, *p* > 0.05). **F.** EM of *D*. *discoideum* infected with *M*. *marinum* wt for 7 h and incubated or not with PI at 2.5× for an additional hour. White and green asterisks label bacteria and autophagosomes, respectively. White arrowheads point to membrane extensions originating at the MCV. The black arrowhead marks the zoomIn. Scale bars, 2 μm.

To test the hypothesis of *M*. *marinum* blocking the autophagic flux in an ESX-1-dependent manner, we measured the flux using an improved protocol tailored to the specific infection conditions (see Materials and Methods and S5 Fig). Infection with *M*. *marinum* wt but not ΔRD1 led to a general increase in the number of cytoplasmic GFP-Atg8^+^ structures (Fig 6A), but treatment with a protease inhibitor (PI) cocktail, which impairs lysosomal function (S6A Fig) but does not alter the amount of cytosolic GFP-Atg8 (S6B Fig), resulted in the accumulation of such structures in both infected and non-infected cells (Fig 6A and 6B). However, this accumulation was less apparent in cells infected with *M*. *marinum* wt compared to ΔRD1 or to uninfected cells (Fig 6A–6C). Despite the increased numbers of GFP-Atg8 puncta, by immunoblot, the free GFP signal was reduced in cells infected with both wt and ΔRD1 bacteria at 1.5 and 7 hpi (Fig 6D and 6E). Both the induction and the blockade of the autophagic flux can lead to a decrease in the free GFP signal in western blot (S5 and S6C and S6D Figs) [30], but because the ratio in GFP-Atg8 puncta between cells treated with PI and not treated was lower for an infection with *M*. *marinum* wt (Fig 6C), we conclude that only *M*. *marinum* wt blocks the autophagic flux. Nevertheless, this ratio was not equal to one, suggesting again the parallel induction of autophagosome formation by *M*. *marinum* wt. The ESX-1-dependent blockade of the autophagic flux as well as the induction of autophagy by *M*. *marinum* were confirmed by treatment with the vacuolar H^+^-ATPase inhibitor concanamycin B (CMB) (see Materials and Methods and S7 Fig).

The hypothesis that *M*. *marinum* wt blocks the autophagic flux is supported by the presence of membrane tubules or lamella projecting from the MCV, which appear more prominent after PI treatment (Figs 6F and S8A). These resemble the proto-lysosomal tubules generated during autophagic lysosomal reformation (ALR) [44, 45] or omegasomes [4]. The accumulation of undigested membranes and cytoplasmic material inside the MCV, observed by EM even after treatment with AR-12 (S8A Fig), also supports the view of a block induced by *M*. *marinum*.

The requirement for a functional ESX-1 secretion system for both induction of autophagosomes and blockade of the autophagic flux was confirmed by the fact that PI treatment only increased the accumulation of GFP-Atg8 around wt but not ΔRD1 bacteria (S8B Fig**)**. In addition, we observed ΔRD1 bacteria completely surrounded by a GFP-Atg8^+^ vacuole only after PI treatment, while it did not change the percentage of *M*. *marinum* wt inside such structures (S8C Fig). This indicates that killing and degradation of *M*. *marinum* ΔRD1 by autophagy is more prominent than for *M*. *marinum* wt, and that ESX-1 is essential to block the flux. Altogether, our data suggest that, in *D*. *discoideum*, *M*. *marinum* induces the formation of autophagosomes while it blocks the autophagic flux in an ESX-1-dependent manner.

### The early autophagic response induced by *M*. *marinum* might depend on TORC1

**Fig 7:**
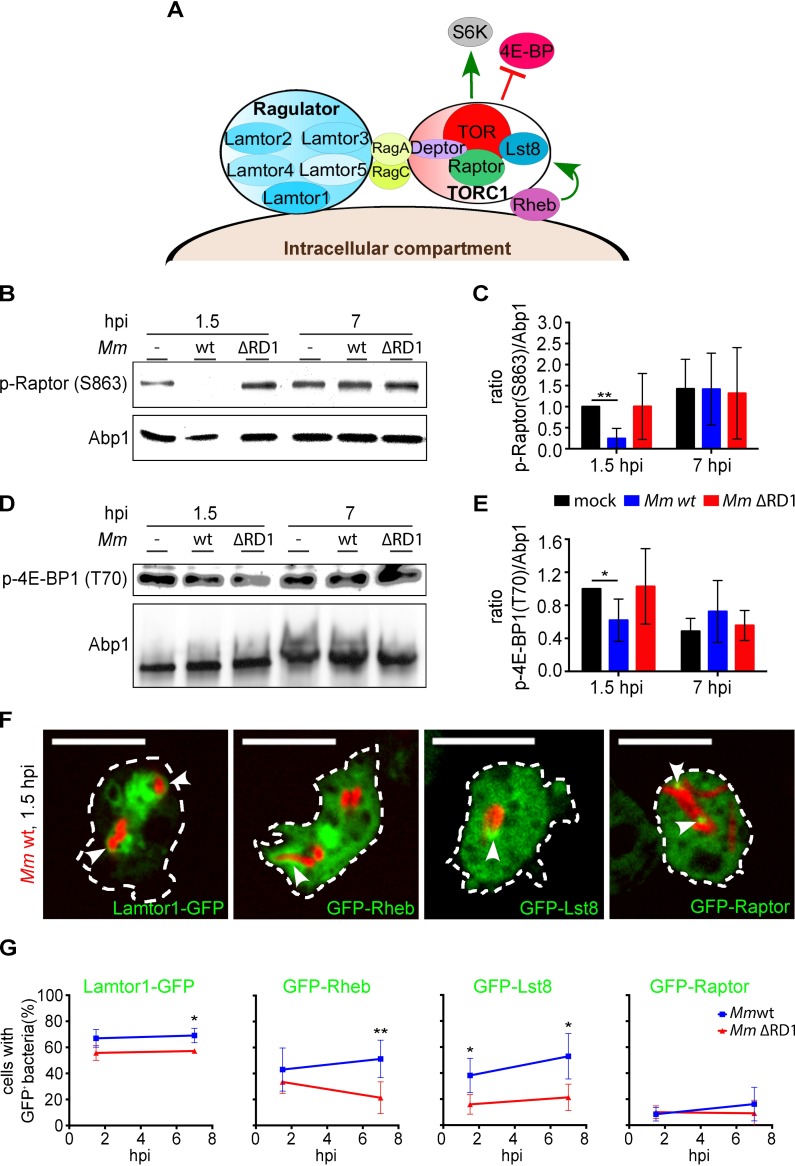
The early autophagic response, but not the flux blockade induced by *M*. *marinum* depends on TORC1. **A.** Scheme of the putative TORC1 and Ragulator complexes in *D*. *discoideum*. Only the proteins conserved in the amoeba are represented. Green arrows indicate activation; red lines, inhibition; **B and D.** *D*. *discoideum* was infected or not with mCherry-expressing *M*. *marinum* wt or with DsRed-expressing *M*. *marinum* ΔRD1 for 1.5 and 7 hpi and immunoblotted against p-Raptor(S863) (**B**), and p-4E-BP1(T70) (**D**). Abp1 was used as loading control. Shown are representative results from four independent experiments; **C and E.** Mean and standard deviation of the ratio p-Raptor(S863)/Abp1 (**C**) and p-4E-BP1(T70)/Abp1 (**E**) from the immunoblots represented in **B** and **D**. Unpaired *t* test (*p ≤ 0.05; **p ≤ 0.01); **F.** Sections of live cells expressing Lamtor1-GFP, GFP-Rheb, GFP-Lst8 or GFP-Raptor and infected with mCherry-expressing *M*. *marinum* wt for 1.5 h. White arrowheads point to the recruitment of the GFP-constructs to the MCV. Scale bars, 10 μm; **G.** Quantification of the percentage of cells carrying mCherry-expressing *M*. *marinum* wt or DsRed-expressing *M*. *marinum* ΔRD1 positive for Lamtor1-GFP, GFP-Rheb, GFP-Lst8 or GFP-Raptor at 1.5 and 7 hpi. Mean and standard deviation from three to six independent experiments. A minimum of 100 infected cells were counted for each cell line. Unpaired *t* test compared wt and ΔRD1 bacteria (*p ≤ 0.05; **p ≤ 0.01).

The main inhibitor of autophagy is TOR, a highly-conserved kinase that associates with Raptor, Lst8 and Deptor to form the TORC1 complex (Fig 7A). Some pathogenic bacteria manipulate the autophagic defence response in host cells via regulation of TORC1 activity [40]. Therefore, we investigated whether *M*. *marinum* also controls autophagy in *D*. *discoideum* by impacting on TORC1.

As a readout for TORC1 activity, we monitored the phosphorylation of conserved sites in Raptor and in the TOR substrate 4E-BP1 during infection (see Materials and Methods and Fig 7B–7E). At 1.5 hpi, TORC1 activity was decreased, since both p-Raptor (Fig 7B and 7C) and p-4E-BP1 (Fig 7D and 7E) were significantly reduced. This inhibition of TORC1 activity was transient, and at 7 hpi the phosphorylation levels of both proteins returned to control levels (Fig 7B–7E). Antibodies against mammalian 4E-BP1 or Raptor did not detect the endogenous proteins in *D*. *discoideum*, but we confirmed by monitoring the level of GFP-Raptor that the inhibition of TORC1 activity was due to the decrease in p-Raptor and not to its degradation (S9 Fig). For unknown reasons, the results obtained during infection with the ΔRD1 bacteria were very variable, impeding a definite conclusion on whether the ESX-1 system was responsible for the regulation of TORC1 during *M*. *marinum* infection.

In mammalian cells, TORC1 translocates from the cytosol to membranes of late endosomes and lysosomes upon activation by amino acids [46], while it always resides at the vacuolar membrane in yeasts [47]. Since the MCV in *D*. *discoideum* possesses characteristics of late endosomes (Fig 5), we monitored whether TORC1 was also located on bacteria compartment (Fig 7F). Recruitment of GFP fusions of Lst8 and Raptor to the MCV was observed at 1.5 hpi. We also detected recruitment of the lipid-anchored membrane proteins Lamtor1 and Rheb (Fig 7A), which activate TORC1 when amino acid levels are high [48]. However, unlike TORC1 activity, the recruitment of all these proteins to the MCV did not significantly change between 1.5 and 7 hpi (Fig 7G), indicating that recruitment is probably independent of TORC1 activity. Nevertheless, we detected a significant decrease in the recruitment of Lamtor1, Rheb and Lst8 to the *M*. *marinum* ΔRD1 MCV at 7 hpi (Fig 7G), suggesting that the features of the bacterial compartment diverge during infection depending on the functionality of the ESX-1 system.

We conclude that, early after infection of *D*. *discoideum*, *M*. *marinum* induces autophagosome formation presumably via downregulation of TORC1, which might be dependent on the activity of the ESX-1 secretion system. However, this downregulation is not associated with changes in the localization of TORC1, which remains associated with the MCV at all times.

## Discussion

In recent years, the professional phagocyte *D*. *discoideum* has become a powerful model to study host-pathogen interactions at the cellular level [23, 49]. The functional conservation of the autophagy pathway between *D*. *discoideum* and mammalian cells, in addition to its genetic tractability, make this amoeba an excellent model system to uncover the mechanisms of xenophagy [24]. In this study, we validate the infection model *D*. *discoideum*-*M*. *marinum* as a robust system to uncover the complex manipulation of autophagy by mycobacteria.

It was previously shown that *M*. *marinum* recruits LC3/Atg8 in murine macrophages and zebrafish embryos [21, 50]. In *D*. *discoideum*, we detected not only an increase in the number of Atg8^+^ and Atg18^+^ structures (Figs 1A and 1B and S1D–S1E), but the recruitment of those structures and other autophagy markers like Ub and p62 to the MCV early after infection (Fig 1C, 1D and 1F). This recruitment is likely due to an early induction of autophagosome formation, since the activity of the main autophagy inhibitor, TORC1, decreased at 1.5 hpi (Fig 7B–7E), the transcription of some of the genes involved in nucleation (*atg1*) and elongation (*atg8*) of the isolation membrane, the two major steps in autophagosome formation [51, 52], were upregulated at the beginning of the infection (Fig 1G), and translocation of GFP-Atg8 from the cytosol to autophagosomal membranes can also be observed from 1.5 hpi (Figs 4F, 1A and 1B). With time during infection (5–7 hpi), the autophagy induction driven by *M*. *marinum* diminished, as confirmed by the decrease in (1) the transcription of autophagy genes (Fig 4E), (2) the total number of GFP-Atg8 structures per cell (*Mm* wt mock in Figs 6B and S7D), and (3) the recruitment of those GFP-Atg8^+^ and GFP-Ub^+^ structures to the MCV (Fig 4A and 4B). Intriguingly, at 24 hpi transcription of autophagy genes was no longer upregulated (Fig 4E), even though the recruitment of Atg8 to the bacteria raised back to the initial level (Fig 4A). We have recently shown that *M*. *marinum* recruits the autophagic machinery during its ejection from *D*. *discoideum*, a process that enables the non-lytic cell-to-cell transmission of the bacterium after 24 hpi [25]. Our results suggest that, later in the infection, *M*. *marinum* benefits from pre-formed autophagosomes for its ejection from the host cell.

Surprisingly, at early stages only 5–10% of the co-localization events between bacteria and Atg8 corresponded to closed compartments (Fig 1D), suggesting that only a few mycobacteria are captured in autophagosomes. Autophagy is reported to play both negative and positive roles during infection. When it acts as a cell-autonomous defence response, it leads to bacterial killing, but it can also support replication of pathogens such as *S*. *aureus* or *Brucella abortus* [5]. Despite inducing an early autophagic response, *M*. *marinum* not only survives but proliferates inside *D*. *discoideum* (Fig 2D, mock). This points to a complex interface between *D*. *discoideum* and *M*. *marinum* in which autophagy plays both a negative and a positive role. We suggest that overall autophagy restricts bacterial load, as revealed by the unrestricted proliferation in *atg1*- and *atg8*- cells (Figs 3A and S3A). This loss of control of the mycobacteria replication can only be due to autophagic deficiency and not to the lack of an inflammatory response, as previously suggested to occur in mice [53], since *D*. *discoideum* lacks a canonical inflammation pathway. However, in these mutant cells, the bacteria escape precociously to the cytosol (Fig 3B) due to a compromised integrity of the MCV, indicating a contribution of autophagy to the maintenance/repair of damaged MCV membranes early during infection. A similar cyto-protective role of autophagy in repairing membrane damages caused by *Salmonella* has been suggested [54]. In addition, a role of the ESX-1 secretion system in the induction of autophagy in animal cells has been reported [21, 55]. Here, we suggest that *M*. *marinum* uses the same membrane damage-dependent mechanism to subvert autophagy in the ancient phagocyte *D*. *discoideum*. Note that in wt *D*. *discoideum* the capacity of autophagy to maintain the integrity of the MCV is likely overwhelmed at later stages of infection by *M*. *marinum*, leading to ESX-1-dependent cytosol escape. In any case, a massive physiological or pharmacological induction of autophagy can overcome such positive effects, leading to restriction of mycobacterial load [13, 56], as we also observed in *D*. *discoideum* after treatment with AR-12, AZD8055 and PI-103 (Figs 2D and S2F).

Our observations of autophagosomes associated with the MCV and of regions of double membrane at the periphery of the MCV (green arrowheads in S8A Fig) suggest an additional or alternative role for autophagy in supplying the MCV with membranes. The formation of MCV with features of autophagic membranes was completely dependent on a functional ESX-1 secretion system, since deletion of the RD1 locus tremendously reduced the recruitment of Atg8 and Ub to the damaged MCV (Fig 4A–4C) and abolished the upregulation of autophagy genes (Fig 4E). We confirmed that the decrease in the percentage of cells with GFP-Atg8^+^ bacteria observed during infection with *M*. *marinum* ΔRD1 (Fig 4A) was not due to a lower bacterial load (Fig 4D), since quantifying the percentage of mutant bacteria positive for GFP-Atg8 showed a similar reduction (S4C Fig). In addition, the attenuation of *M*. *marinum* ΔRD1 observed in wt *D*. *discoideum* was not epistatically reversed by a knock out of the autophagy pathway (Fig 4D), likely indicating that xenophagy mainly limits cytosolic bacteria burden.

To impede its killing by xenophagy, *M*. *marinum* must somehow block the autophagic flux. We show here that, in *D*. *discoideum* as in macrophages [21], *M*. *marinum* resides in a compartment with late endosomal features but devoid of lysosomal properties (Fig 5). A role for ESX-1 in flux blockade has been proposed for *M*. *tuberculosis* [13]. Here, we demonstrate for the first time that *M*. *marinum* escapes from lysosomes and also blocks the autophagic flux in an ESX-1-dependent manner (Figs 5I, 5J, 6B–6E, S7D–S7E and S8C). The membrane perforations caused by the ESX-1 secretion system might alter the content or properties of the MCV, preventing its fusion with lysosomes, as previously shown for the action of Listeryolisin O from *Listeria monocytogenes* [57, 58]. Actually, *M*. *marinum* ΔRD1 does not induce autophagosome formation or recruitment (Figs 4A–4C, 4E and 6A, mock) nor blocks the autophagic flux (Figs 6B, 6C and S7D–S7E). However, the decrease in cytosolic GFP-Atg8 fluorescence at 1.5 hpi (Fig 4F), the decrease in free GFP (Fig 6D) and the increase of bacteria inside autophagosomes after PI treatment (S8C Fig) when *D*. *discoideum* is infected with *M*. *marinum* ΔRD1 suggest that it might undergo enhanced autophagic clearance. Altogether, our data indicate that *M*. *marinum* utilizes the ESX-1 secretion system to block the autophagic flux in *D*. *discoideum* and to tailor the composition of the MCV.

Little is known about the role of TOR during mycobacterial infections. Mycobacterial lipids and ESAT-6 stimulate TOR signalling [59, 60], but the kinetics of TOR activity and autophagy during infection have not been studied. Here, we show that early after infection, *M*. *marinum* inhibits TORC1 activity, which is reactivated at 7 hpi (Fig 7B–7E). TORC1 reactivation might contribute to the block of autophagic flux. In agreement with this, at 8 hpi, the MCV was full of cytoplasmic material, and membrane tubules (reminiscent of ALR) or lamella extended from the compartment (Fig 6F). ALR involves the autophagy-dependent reactivation of TOR after inhibition by stress, and consists in the formation of proto-lysosomal tubules from autolysosomes that finally mature into functional lysosomes [44, 45]. On the other hand, these membrane extensions or lamella also resemble omegasomes arising from the compartment containing *M*. *marinum*, which is supported by the localization of GFP-Atg18 (S1D–S1E Fig) at the MCV. This suggests the MCV as a site for autophagosome formation, but more experiments will be necessary to confirm the origin of those membrane tubules/lamella at the MCV.

**Fig 8:**
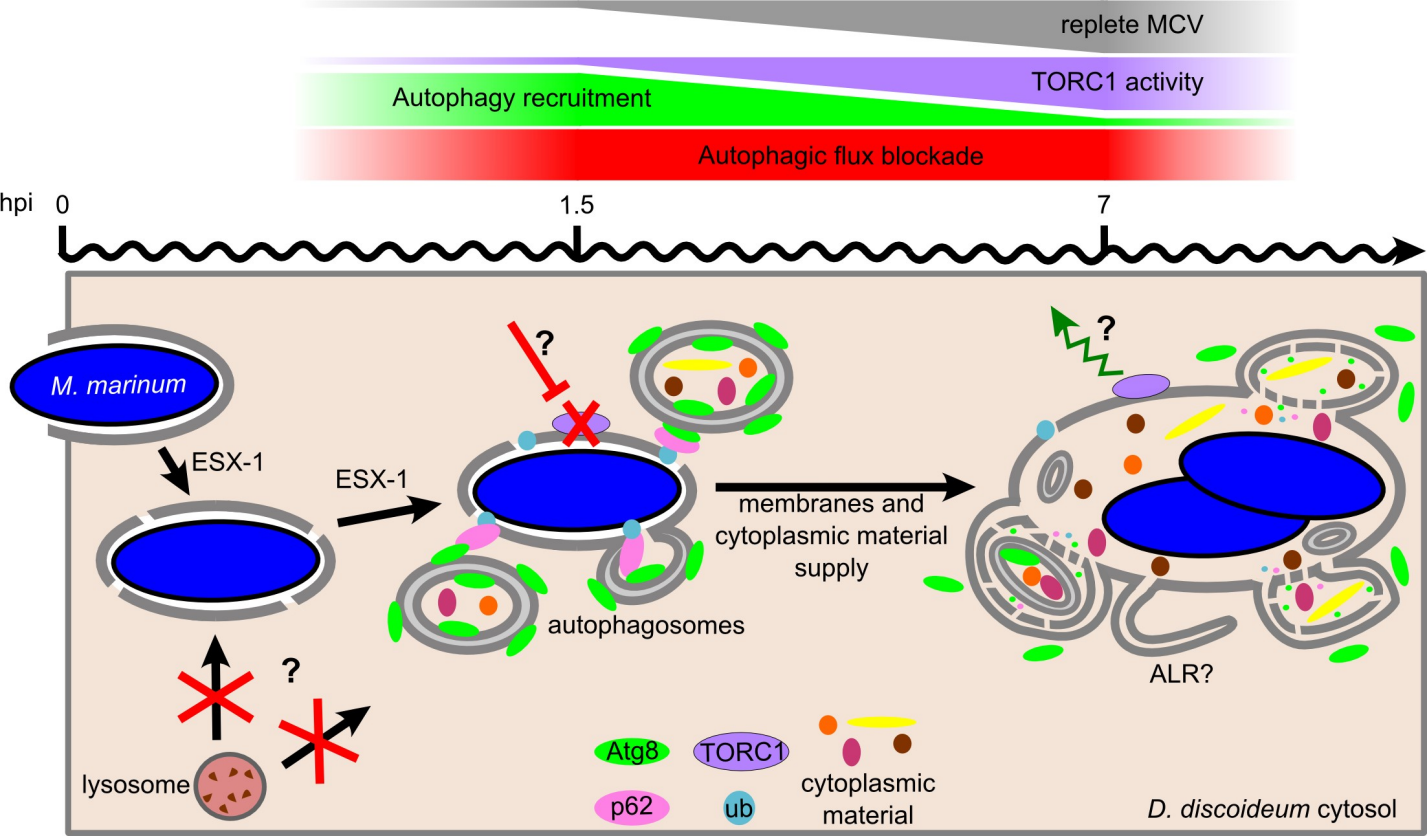
Model of the *D*. *discoideum* autophagic response during *M*. *marinum* infection. Early after engulfment, *M*. *marinum* damages the membrane of its MCV in an ESX-1-dependent manner. Membrane perforations might block lysosome fusion and, consequently, autophagic flux. In addition, ESX-1 inhibits TORC1 activity early during infection (1.5 hpi), presumably through a nutrient-sensing pathway as described for other bacteria [75–77]. Downregulation of TORC1, which is always bound to the wt MCV membrane, induces the formation of autophagosomes that somehow repair the membrane damages and provide the MCV with cytoplasmic material. TORC1 re-activation at 7 hpi leads to the decrease in autophagosome formation and recruitment to the MCV, and enhances the blockade of the autophagic flux, which generates proto-lysosomal tubules (ALR). The block in autophagic flux prevents bacteria killing in autolysosomes and degradation of membranes and cytoplasmic material delivered to the MCV via the recruited autophagosomes.

In conclusion, we propose that the membrane damages caused by the *M*. *marinum* ESX-1 secretion system activate an early cell-autonomous defence response in *D*. *discoideum*, which is accompanied by the transient inhibition of TORC1 activity. However, *M*. *marinum* avoids killing inside induced autolysosomes by blocking the autophagic flux, likely as a consequence of the membrane perforations. The autophagic flux blockade results in the accumulation of membranes and cytoplasmic material in the MCV, which might somehow support bacterial survival within the niche (Fig 8).

## Materials and methods

### *Dictyostelium* strains and culture

*The D*. *discoideum* strains used for this work are listed in S1 Table. Cells were axenically grown at 22°C in HL5c medium (Formedium) supplemented with 100 U mL^−1^ of penicillin and 100 μg mL^−1^ of streptomycin (Invitrogen).

To generate the *p62* knockout strain, *p62* was amplified with the primers p62F10 and p62R10 from genomic DNA of DH1 and ligated into pGEM-T-Easy (Promega). The Bs-resistance cassette flanked by SmaI sites from plasmid pLPBLP [61] was inserted into pGEM-T-p62, previously digested with *Eco*RV and *Bcl*I and blunted.

Plasmids and primers to generate the *D*. *discoideum* GFP-tagged proteins Atg8, Atg18, Atg1, Ub, p62, Rab11c, Rab7a, VatB, Lamtor1, Rheb, Lst8 and Raptor are listed in S1 and S2 Tables. Plasmids were transfected into *D*. *discoideum* by electroporation and selected with the relevant antibiotic. Hygromycin and G418 were used at a concentration of 50 and 5 μg mL^−1^, respectively.

### Mycobacteria strains and culture

Mycobacteria, listed in S1 Table, were cultured in 7H9 (Difco) supplemented with 10% OADC (Becton Dickinson), 0.2% glycerol and 0.05% Tween80 at 32°C in shaking culture at 150 r.p.m. To minimize bacterial aggregation, Erlenmeyer flasks containing 5 mm glass beads were used. Cultures were grown until OD_600_ of 1 (1.5 x 10^8^ bacteria mL^−1^). *M*. *marinum* ΔRD1 was complemented with the RD1-2F9 cosmid by integration into the chromosomal *attB* site [42].

### Bacteria growth measurements

Growth of luminescent bacteria was measured as described previously [62]. Briefly, 1.0 × 10^5^ *luxABCDE*-expressing *M*. *marinum* were plated on a non-treated, white F96 MicroWell plate (Nunc) containing 7H9-OADC medium and covered with a gas permeable moisture barrier seal (Bioconcept). Luminescence was measured for 30–70 h with 1 to 3 h intervals with a Synergy Mx Monochromator-Based Multi-Mode Microplate Reader (Biotek). The temperature was kept constant at 32°C. For intracellular growth measurements, dilutions between 0.5 and 2.0 × 10^5^ of infected cells were plated as described above with HL5c medium containing 10 μM of amikacin to avoid extracellular growth of mycobacteria. Luminescence was also measured for 30–70 h with 1 h intervals at a constant temperature of 25°C.

### Cell cytotoxicity assay

Cell cytotoxicity was determined as the percentage of reduction of the signal from *alamarBlue* reagent, as indicated by the manufacturer (Invitrogen). Briefly, 2.0 × 10^5^ cells were cultured on a 96-well plate with HL5c containing DMSO or AR-12 for 4 h at 21°C. After washing with phosphate buffer, cells were cultured in the dark with 10% (v/v) *alamarBlue* reagent for an additional 4 h. Finally, fluorescence at 595 nm was measured and normalized to 1 (DMSO carrier).

### Antibodies, reagents, immunoblot and immunofluorescence microscopy

As readouts of TORC1 activity [63, 64], the rabbit polyclonal antibody (pAb) anti-p-Raptor(S863) (Santa Cruz Biotechnology) and the rabbit pAb anti-p-4E-BP1(T70) (Cell Signaling Technology), which recognise the conserved phosphorylated sites in *D*. *discoideum*, were used. A rabbit pAb was raised against full-length recombinant Atg8; anti-p80 [65] was purchased from the Geneva Antibody Facility (http://www.unige.ch/antibodies); the anti-Ub(FK2) monoclonal antibody (mAb) was from Enzo Life Sciences; rabbit anti-Abp1 pAb was previously described [66]; mAb and pAb anti-GFP were from Abmart and MBL, respectively; rat mAb anti-RFP was from ChromoTek; rabbit Goat anti-mouse or anti-rabbit IgG coupled to Alexa488 or Alexa594 (Invitrogen) or to HRP (Brunschwig) were used as secondary antibodies.

To stain live bacteria, 10 μM Vibrant DyeCycle Ruby Stain (ThermoFisher) was added to the infection sample 30 min before imaging. To stain acidic compartments, 1 μM LysoSensor Green DND-189 (ThermoFisher), a pH-dependent probe which fluorescence intensity increases upon acidification, was added to the infected cells. After 10 min incubation, excess dye was washed away and cells were imaged for a maximum of 30 min. To stain compartments with proteolytic activity, 50 μg/mL DQ Green BSA (ThermoFisher), which releases fluorescent protein fragments upon dequenching of the self-quenched, BSA-associated Bodipy dye by proteolysis, was added to the infection sample one hour before imaging. To measure the autophagic flux, the cOmplete EDTA-free PI cocktail from Roche (11873580001) and CMB (BioViotica) were used. To assess inhibition of the lysosomal function, before treatment with PI and CMB (1 h and 2 h, respectively) cells were incubated overnight in the dark with 4 mg/mL TRITC-Dextran (Sigma) and 0.4 mg/mL FITC-Dextran (Sigma). To prepare lysates for immunoblotting, the cOmplete, ULTRA Tablets, Mini, *EASYpack* PI cocktail from Roche (05892970001) were used. PhosSTOP phosphatase inhibitor cocktail was also from Roche (04906837001). AR-12 (OSU-03012), AZD8055 and PI-103 were purchased from Selleckchem.

For immunoblotting of samples to monitor the autophagic flux and TORC1 activity, 3.6 × 10^6^ cells were gently collected in a 15 mL Falcon conical tube on ice and centrifuged 5 min at 1500 r.p.m. in the cold. The pellet was resuspended in RIPA buffer (Tris 20 mM, NaCl 50 mM, sodium deoxycholate 0.5%, NP-40 1%, pH 7.4) containing protease and phosphatase inhibitors, and lysis was allowed for 10 min on ice. Lysates were then centrifuged 10 min at 13000 r.p.m. in the cold and boiling sample buffer added to the supernatant at 2×. Samples were further boiled at 95°C for 5 min. Final cell density in the sample was 5 × 10^7^ cells mL^−1^. Around 5 × 10^5^ cells were loaded per lane.

After SDS-PAGE separation [67] and transfer onto nitrocellulose membranes (Protran, Schleicher & Schuell), immunodetection was performed as previously described [68] but with ECL Prime Blocking Reagent (Amersham Biosciences) instead of non-fat dry milk. When the GFP-Atg8 cleavage was analysed, a mix of mAb and pAB anti-GFP was used to enhance the signal. Detection was performed with ECL Plus (Amersham Biosciences) using an EpiChemi II Darkroom device (UVP). Data quantification was carried out with ImageJ.

Infected *D*. *discoideum* cells were fixed by rapid freezing in ultracold methanol and immunostained as previously described [69]. Immunofluorescence images were recorded either with a Leica SP5 or Zeiss LSM700 or LSM780 confocal microscope using a 63× 1.4NA or a 100× 1.4NA oil-immersion objective.

### Infection assay

Infections were performed as previously described [35], with few modifications. Briefly, after infection and phagocytosis, extracellular bacteria were washed off and attached infected cells were resuspended in HL5c containing 5 μg mL^−1^ of streptomycin, 5 U mL^−1^ of penicillin.

### Live imaging

Cells were plated on a μ-dish (iBIDI) at the indicated times and, after adherence, either 1μm sections or time-lapse movies with 4 s intervals were taken with a spinning disc confocal system (Intelligent Imaging Innovations) mounted on an inverted microscope (Leica DMIRE2; Leica) using the 63 × or the 100 × 1.4 NA oil objective. Images were processed with ImageJ. Quantification of autophagic fluorescent structures, bacteria co-localization or cytosolic GFP-Atg8 fluorescence intensity was performed manually. Under infectious conditions, only cells containing bacteria were considered for quantification. To improve imaging in S1A–S1C Fig, a 1 mm thin agarose sheet was overlayed in the middle of the μ-dish as described previously [70].

### Electron microscopy (EM)

EM in Figs 3B and S2B and S2C was performed as previously described [71]; For Figs 1E, 6F and S8A, the exact protocol described before [72] was followed. Samples were processed and analysed at the EM Core facility of the Faculty of Medicine (University of Geneva).

### Quantitative real-time PCR (qPCR)

RNA from non-infected cells or cells infected with *M*. *marinum* wt or ΔRD1 at appropriate times was extracted using the Direct-zol RNA MiniPrep kit (Zymo Research) as indicated by the manufacturer. 1 μg RNA was retro-transcribed using the iScript cDNA Synthesis Kit and polydT primers (Biorad). The cDNA was amplified using the primers listed in S2 Table and the SsoAdvanced universal SYBR Green supermix (Biorad). Amplimers for *atg1*, *atg8a*, *atg8b*, *p62* and *gapdh* were detected on a CFX Connect Real-Time PCR Detection System (Biorad). The housekeeping gene *gapdh* was used for normalization. PCR amplification was followed by a DNA melting curve analysis to confirm the presence of a single amplicon. Relative mRNA levels (2^−ΔΔCt^) were determined by comparing first the PCR cycle thresholds (Ct) for the gene of interest and *gapdh* (ΔC), and second Ct values in infected cells vs non-infected cells (ΔΔC).

### Autophagic flux assay

Current autophagic flux assays in *D*. *discoideum* rely on a 4 h treatment with the lysosomotropic compound NH_4_Cl to suppress lysosomal acidification [73], which induces adverse effects on cell and organelle morphology. We therefore, developed an alternative assay using a cocktail of PI to block autolysosomal proteolysis of GFP-Atg8 (S5 Fig). Because of the fast autophagic flux in *D*. *discoideum* (S1A–S1C Fig) [30], as short as one hour of treatment was required to detect a lack of lysosomal function (S6A Fig) and an accumulation of GFP-Atg8 by immunoblot (S6C and S6D Fig). In addition, we also observed a 2-fold accumulation of GFP-Atg8-positive structures by live microscopy (S6E and S6F Fig). As a positive control for the flux assay, we used AR-12, which increased the number of autophagosomes (S6E and S6F Fig), as well as the rate of degradation of the GFP cleaved from GFP-Atg8 (S6C and S6D Fig). The induction of flux was confirmed by the detection of 2.5-fold more GFP-Atg8 puncta in cells treated with AR-12 and PI compared to cells treated only with AR-12 (S6E and S6F Fig). These induction and blockade of the autophagic flux by AR-12 and PI, respectively, were not toxic, since the cell shape remained unaltered (S6E Fig). We confirmed that these effects were autophagy-dependent, since almost no GFP-Atg8 cleavage or accumulation were observed in treated *atg1*- cells (S6C–S6F Fig).

Infected or non-infected cells expressing GFP-Atg8 were cultured in a shaking Falcon 6 Well Clear Multiwell Plate at a density of 1.33 × 10^6^ cells mL^−1^. One hour before the indicated time point, the PI cocktail was added at 2.5-fold the recommended concentration. Addition of ddH_2_O was used as mock-treatment. As a positive control, AR-12 was added 2 h before the indicated time point (1 h before the PI treatment) at a final concentration of 2.5 μM. In this case, DMSO was used as a mock-treatment. Cells were collected either on ice for immunoblotting or on a μ-dish for live imaging (see above).

Similar results were obtained with the vacuolar H^+^-ATPase inhibitor CMB (S7 Fig). Two-hours treatment with 1 μM CMB (or DMSO as mock-treatment) was needed for a clear impairment of the lysosomal function (S7A Fig) and the autophagic flux (S7B and S7C Fig). However, since 2 h, but not 1 h, treatment mildly but significantly affected *atg1*- cells (S7C Fig), 1.5 h treatment with CMB was finally used to monitor the autophagic flux during *M*. *marinum* infection (S7D–S7E Fig).

### Statistical analysis

The GraphPad Prism and MATLAB software were used to perform statistical tests and to plot graphs. For the autophagic flux assays (Figs 6B, S6F and S7C and S7D), all data sets were fitted to Poisson distributions, and the corresponding parameter λ was estimated. 10^6^ random permutations of the data sets were performed to generate random λ. These λ were used to calculate the probability of differences occurring by chance between the pairs of λ of the original data sets, as described in [74]. The λ of each data set and the corresponding *p*-values obtained by the random permutation test are listed in S3 and S4 Tables, respectively. In Figs 6C and S7E, the log_2_ of the ratios between the λ in drug-treated (PI or CMB) and mock-treated cells was calculated. The probability of differences occurring by chance were similarly tested using 2x10^6^-random permutations of the data sets.

## Supporting information

S1 References Supporting references. (DOCX)

S1 Fig GFP-Atg18 early response to *M*. *marinum* infection in *D*. *discoideum*. **A.** Video frames showing the formation and degradation of one GFP-Atg8^+^ autophagosome. Scale bars, 1 μm; **B.** Video frames of two more examples of GFP-Atg8^+^ autophagosomes flux in *D*. *discoideum*. Scale bars, 1 μm; **C.** Time-lapse captures of one fusion event among GFP-Atg8^+^ vesicles in *D*. *discoideum*. Scale bars, 1 μm; **D.** Two representative maximum projections of GFP-Atg18-expressing *D*. *discoideum* cells infected (right) or not (left) with mCherry-expressing *M*. *marinum* wt. Images were recorded live 1.5 h after infection. White arrowheads point to GFP-Atg18 structures. Scale bars, 5 μm; **E.** Median and interquartile ranges of the number of GFP-Atg18 structures per cell at 1.5 hpi. 57–97 cells from two independent experiments were counted. Unpaired *t* test (****p ≤ 0.0001).
(PDF)

S2 Fig Manipulation of autophagy in *D*. *discoideum* by AR-12. **A.** Representative maximum projections of live GFP-Atg8-expressing *D*. *discoideum* treated or not with AR-12 at 2.5 μM for 2 hours. Scale bars, 10 μm; **B.** EM of GFP-Atg8-expressing *D*. *discoideum* cells treated or not with AR-12 at 5 μM for 1 hour. Nuclei are labelled by the letter <N>. White arrowheads label large double membrane compartments engulfing cytosolic material. Scale bars, 1 μm; **C.** Reconstruction by EM of the autophagosome formation events in *D*. *discoideum* after one hour incubation with AR-12 at 5 μM: 1. nucleation; 2–6. elongation; 7. closure; 8. maturation. Scale bars, 0.4 μm; **D.** Cell cytotoxicity of *D*. *discoideum* treated or not with AR-12 at 2.5 μM for 4 h. Mean and standard deviation of three experiments; **E.** AR-12 at 2.5 μM was added or not to *M*. *marinum* and the bacterial growth was monitored (RLU) in triplicates; **F.** Cells infected with *lux*-expressing *M*. *marinum* wt bacteria were treated or not with AZD8055 or PI-103 at 2.5 μM. Intracellular bacterial growth (RLU) is represented as the mean and standard deviation from duplicates.
(PDF)

S3 Fig *M*. *marinum* proliferation in *D*. *discoideum* autophagy mutants. **A.** *D*. *discoideum* wt, *atg8*- and *p62*- cells were infected with *lux*-expressing *M*. *marinum* wt and intracellular bacteria growth was measured (RLU). One representative experiment of three; **B.** *D*. *discoideum atg1*- cells were infected with *lux*-expressing *M*. *marinum* wt and treated or not with AR-12 at 2.5 μM. The intracellular bacterial growth was monitored as RLUs. The average of the RLUs from three consecutive time points, in triplicates, is represented.
(PDF)

S4 Fig Cytosolic *M*. *marinum* are ubiquitinated in *D*. *discoideum*. **A.** *D*. *discoideum* was infected with mCherry-expressing *M*. *marinum*, fixed and stained against p80 (red), Ub (green) and mCherry (blue). Representative maximum projections at 24 hpi. White arrowheads point to ubiquitination. Scale bars, 10 μm (upper panel) and 2 μm (panels 1 and 2); **B.** *D*. *discoideum atg1*- Atg1-GFP cells were infected with mCherry-expressing *M*. *marinum*, fixed and stained for Ub (green) and mCherry (red). Representative maximum projections at 0.25 and 6 hpi. Scale bars, 10 μm. **C.** Infections represented in Fig 4A were also quantified as the percentage of *M*. *marinum* wt and ΔRD1 (expressing mCherry and DsRed, respectively) positive for GFP-Atg8 at 1.5, 7 and 24 hpi. Means and standard deviations from independent triplicates. A mean of 171 and 93 MCVs per time point was counted for *M*. *marinum* wt and ΔRD1 infection, respectively; **D.** *D*. *discoideum atg1*- was infected with *M*. *marinum* wt and ΔCE (both expressing mCherry), fixed and stained for Ub (green) and mCherry (red). Quantification of the percentage of bacteria (red) positive for Ub (green) at 6 hpi.
(PDF)

S5 Fig PI-based autophagic flux assay in *D*. *discoideum*. Scheme of the autophagic flux assay in *D*. *discoideum* expressing exogenous GFP-Atg8 and the expected results. In mock conditions autophagosomes form and fusion with lysosomes, leading to the degradation of the engulfed cytoplasmic material and the GFP-Atg8 protein at the inner membranes. Atg8 is sensitive to degradation whereas GFP, which fluorescence is quenched by low pH, is relatively resistant to hydrolysis [30]. Therefore, after autolysosome formation low signal is detected by fluorescence microscopy while free GFP appears in immunoblotting; Treatment with AR-12 induces both autophagosomes formation and autophagic degradation, while the rates of GFP-Atg8 production in the cell remains the same. As a result, more autophagosomes can be observed by microscopy while the intensity of the free GFP band in immunoblotting is reduced; Short-treatment (1 h) with PI derives in the accumulation of autophagosomes which content cannot be degraded. Hence, by both microscopy and immunoblot higher GFP-Atg8 signal will be observed. Double treatment with AR-12 and PI arises the accumulation of autophagosomes; In *atg1*- cells, autophagosomes cannot be formed under any condition. As a consequence, the GFP-Atg8 signal does not vary during the experiment. It needs to be noticed that some Atg1-independent degradation of GFP-Atg8 may still occur.
(PDF)

S6 Fig PI blocks the autophagic flux in *D*. *discoideum*. **A.** *D*. *discoideum* cells were incubated over night with TRITC-Dextran (pH-insensitive probe that labels the endo-lysosomal pathway in red) and FITC-Dextran (green pH-sensitive probe rapidly bleached upon acidification) followed or not by one hour of treatment with PI at 2.5×. Shown are representative maximum projections of live cells. Increase in the number and/or size of yellow vesicles after PI treatment indicates decrease in the number of acidic compartments. Scale bars, 10 μm; **B.** GFP-Atg8-expressing cells were incubated or not with PI at 2.5× for one hour. Maximum projections were used to measure the IntDen of the cytosolic GFP-Atg8 fluorescence compared to the extracellular IntDen (background). Median with interquartile ranges of the cytosolic GFP-Atg8 IntDen per cell. Each dot represents one cell. More than 350 cells per condition from three independent experiments were counted. Mann-Whitney test (ns, *p* > 0.05); **C.** GFP-Atg8-expressing *D*. *discoideum* wt or *atg1*- cells were treated or not with AR-12 at 2.5 μm. One hour before the end of the treatment, cells were incubated or not with PI at 2.5× and immunoblotted against GFP. Ponceau-S staining was used as loading control; **D.** Ratio GFP/GFP-Atg8 from the immunoblot represented in **C.**; **E.** Representative maximum projections of live GFP-Atg8-expressing *D*. *discoideum* wt and *atg1*- cells under the treatments described in **C.** Scale bars, 10 μm; **F.** Median with interquartile ranges of the number of GFP-Atg8 structures per cell during the treatments carried out in **C.** and **E.** Each dot represents one cell. 116–178 cells per condition were counted. The values of **λ** that define the Poisson distribution of each data set and differences between them were calculated as described in Materials and Methods (**p ≤ 0.01; ****p ≤ 0.0001; ns, *p* > 0.05).
(PDF)

S7 Fig A CMB-based autophagic flux assay confirms the ESX-1-dependent autophagic flux blockade during *M*. *marinum* infection. **A.** *D*. *discoideum* cells were incubated over night with TRITC- and FITC-Dextran followed by 2 h of treatment with 1 μM CMB (DMSO as mock). Shown are representative maximum projections of live cells. Increase in the number and/or size of yellow vesicles after CMB treatment indicates decrease in the number of acidic compartments. Scale bars, 10 μm; **B.** Representative maximum projections of live GFP-Atg8-expressing *D*. *discoideum* wt and *atg1*- cells treated or mock-treated with CMB for 1 or 2 h. Scale bars, 10 μm; **C.** Median with interquartile ranges of the number of GFP-Atg8 structures per cell during the treatments carried out in **B.** Each dot represents one cell. 237–439 cells per condition were counted. The values of **λ** that define the Poisson distribution of each data set and differences between them were calculated as described in Materials and Methods (*p ≤ 0.05; ***p ≤ 0.001; ****p ≤ 0.0001; ns, *p* > 0.05); **D.** GFP-Atg8-expressing cells were infected or mock-infected for 0.5 or 5.5 h with mCherry-expressing *M*. *marinum* wt or with DsRed-expressing *M*. *marinum* ΔRD1 and treated or not with 1 μM CMB for 1.5 additional hours. Medians with interquartile ranges of the number of GFP-Atg8 structures per cell. Each dot represents one cell. 164–551 cells per condition from three independent experiments were counted. The values of **λ** that define the Poisson distribution of each data set and differences between them were calculated as described in Materials and Methods (*p ≤ 0.05; **p ≤ 0.01; ****p ≤ 0.0001; ns, *p* > 0.05); **E.** Mean and standard deviation of the log_2_ (**λ**_CMB_/**λ**_mock_) from the three independent replicates represented in **D**. A log_2_ (**λ**_CMB_/**λ**_mock_) of zero implies that there was a total autophagic block. *p*-values calculated as described in Materials and Methods (*p ≤ 0.05; (****p ≤ 0.0001; ns, *p* > 0.05).
(PDF)

S8 Fig PI and AR-12 treatments during *M*. *marinum* infection. **A.** EM of *D*. *discoideum* cells infected with *M*. *marinum* wt for 6 h and treated or not with AR-12 at 2.5 μm for two additional hours. One hour before the end of this treatment, cells were incubated or not with PI at 2.5×. White, green, red and yellow asterisks label bacteria, phagophores and autophagosomes, pycnosomes, and pycnosomes inside autophagosomes, respectively. White arrowheads point to omegasomes-like membrane extensions; green arrowheads indicate sites of double membranes within the MCV. Nuclei are labelled by the letter <N>. Scale bars, 1 μm; **B and C.** GFP-Atg8-expressing *D*. *discoideum* cells were infected for 5 or 6 h with mCherry-expressing *M*. *marinum* wt or DsRed-expressing *M*. *marinum* ΔRD1 and treated or not with AR-12 at 2.5 μM or PI at 2.5× for 2 or 1 additional hours, respectively. Total time was always 7 hpi. Mean and standard deviation from 2–6 independent replicates of the percentage of cells containing GFP-Atg8^+^ bacteria (**B.**) or the percentage of cells containing bacteria enclosed by a GFP-Atg8^+^ vacuole (**C.**). A minimum of 163 infected cells was counted per condition in **B.**, while 30–258 cells with GFP-Atg8^+^ bacteria were counted in **C.** Unpaired *t* test (***p ≤ 0.001; ****p ≤ 0.0001). nd: not detected.
(PDF)

S9 Fig The decrease in Raptor phosphorylation early after infection of *D*. *discoideum* with *M*. *marinum* wt is not due to Raptor degradation. **A.** GFP-Raptor-expressing *D*. *discoideum* cells were infected or not with mCherry-expressing *M*. *marinum* wt or with DsRed-expressing *M*. *marinum* ΔRD1 for 1.5 and 7 hpi. Representative immunoblots against GFP and Abp1 (loading control) from three independent experiments; **B.** Mean and standard deviation of the ratio GFP-Raptor/Abp1 from the immunoblots represented in **A.**
(PDF)

S1 Table *D*. *discoideum* and mycobacteria strains and plasmids used in this work. (DOCX)

S2 Table Oligonucleotides used in this work. (DOCX)

S3 Table Values of λ defining the Poisson distribution for each data set of GFP-Atg8 structures per cell in the autophagic flux assays. (DOCX)

S4 Table *p*-values obtained by the random permutation test. (DOCX)

## References

[1] Dibble CC, Manning BD. Signal integration by mTORC1 coordinates nutrient input with biosynthetic output. Nat Cell Biol. 2013;15: 555–64. doi: 10.1038/ncb2763 23728461 10.1038/ncb2763 PMC3743096

[2] Sengupta S, Peterson TR, Sabatini DM. Regulation of the mTOR complex 1 pathway by nutrients, growth factors, and stress. Mol Cell. 2010;40: 310–22. doi: 10.1016/j.molcel.2010.09.026 20965424 10.1016/j.molcel.2010.09.026 PMC2993060

[3] Calvo-Garrido J, Carilla-Latorre S, Kubohara Y, Santos-Rodrigo N, Mesquita A, Soldati T, et al Autophagy in Dictyostelium: genes and pathways, cell death and infection. Autophagy. 2010;6: 686–701. 20603609 10.4161/auto.6.6.12513

[4] Carlsson SR, Simonsen A. Membrane dynamics in autophagosome biogenesis. J Cell Sci. 2015;128: 193–205. doi: 10.1242/jcs.141036 25568151 10.1242/jcs.141036

[5] Mostowy S. Autophagy and bacterial clearance: a not so clear picture. Cell Microbiol. 2013;15: 395–402. doi: 10.1111/cmi.12063 23121192 10.1111/cmi.12063 PMC3592990

[6] Steele S, Brunton J, Ziehr B, Taft-Benz S, Moorman N, Kawula T. Francisella tularensis harvests nutrients derived via ATG5-independent autophagy to support intracellular growth. PLoS Pathog. 2013;9: e1003562 doi: 10.1371/journal.ppat.1003562 23966861 10.1371/journal.ppat.1003562 PMC3744417

[7] Schnaith A, Kashkar H, Leggio SA, Addicks K, Kronke M, Krut O. Staphylococcus aureus subvert autophagy for induction of caspase-independent host cell death. J Biol Chem. 2007;282: 2695–706. doi: 10.1074/jbc.M609784200 17135247 10.1074/jbc.M609784200

[8] MacGurn JA, Cox JS. A genetic screen for Mycobacterium tuberculosis mutants defective for phagosome maturation arrest identifies components of the ESX-1 secretion system. Infect Immun. 2007;75: 2668–78. doi: 10.1128/IAI.01872-06 17353284 10.1128/IAI.01872-06 PMC1932882

[9] Simeone R, Bobard A, Lippmann J, Bitter W, Majlessi L, Brosch R, et al Phagosomal rupture by Mycobacterium tuberculosis results in toxicity and host cell death. PLoS Pathog. 2012;8: e1002507 doi: 10.1371/journal.ppat.1002507 22319448 10.1371/journal.ppat.1002507 PMC3271072

[10] Hagedorn M, Rohde KH, Russell DG, Soldati T. Infection by tubercular mycobacteria is spread by nonlytic ejection from their amoeba hosts. Science. 2009;323: 1729–33. doi: 10.1126/science.1169381 19325115 10.1126/science.1169381 PMC2770343

[11] Gutierrez MG, Master SS, Singh SB, Taylor GA, Colombo MI, Deretic V. Autophagy is a defense mechanism inhibiting BCG and Mycobacterium tuberculosis survival in infected macrophages. Cell. 2004;119: 753–66. doi: 10.1016/j.cell.2004.11.038 15607973 10.1016/j.cell.2004.11.038

[12] Castillo EF, Dekonenko A, Arko-Mensah J, Mandell MA, Dupont N, Jiang S, et al Autophagy protects against active tuberculosis by suppressing bacterial burden and inflammation. Proc Natl Acad Sci U S A. 2012;109: E3168–76. doi: 10.1073/pnas.1210500109 23093667 10.1073/pnas.1210500109 PMC3503152

[13] Romagnoli A, Etna MP, Giacomini E, Pardini M, Remoli ME, Corazzari M, et al ESX-1 dependent impairment of autophagic flux by Mycobacterium tuberculosis in human dendritic cells. Autophagy. 2012;8: 1357–70. doi: 10.4161/auto.20881 22885411 10.4161/auto.20881 PMC3442882

[14] Shin DM, Jeon BY, Lee HM, Jin HS, Yuk JM, Song CH, et al Mycobacterium tuberculosis eis regulates autophagy, inflammation, and cell death through redox-dependent signaling. PLoS Pathog. 2010;6: e1001230 doi: 10.1371/journal.ppat.1001230 21187903 10.1371/journal.ppat.1001230 PMC3002989

[15] Mohanty S, Jagannathan L, Ganguli G, Padhi A, Roy D, Alaridah N, et al A mycobacterial phosphoribosyltransferase promotes bacillary survival by inhibiting oxidative stress and autophagy pathways in macrophages and zebrafish. J Biol Chem. 2015;290: 13321–43. doi: 10.1074/jbc.M114.598482 25825498 10.1074/jbc.M114.598482 PMC4505583

[16] Shui W, Petzold CJ, Redding A, Liu J, Pitcher A, Sheu L, et al Organelle membrane proteomics reveals differential influence of mycobacterial lipoglycans on macrophage phagosome maturation and autophagosome accumulation. J Proteome Res. 2011;10: 339–48. doi: 10.1021/pr100688h 21105745 10.1021/pr100688h PMC3018347

[17] Srinivasan L, Gurses SA, Hurley BE, Miller JL, Karakousis PC, Briken V. Identification of a Transcription Factor That Regulates Host Cell Exit and Virulence of Mycobacterium tuberculosis. PLoS Pathog. 2016;12: e1005652 doi: 10.1371/journal.ppat.1005652 27191591 10.1371/journal.ppat.1005652 PMC4871555

[18] Hu D, Wu J, Wang W, Mu M, Zhao R, Xu X, et al Autophagy regulation revealed by SapM-induced block of autophagosome-lysosome fusion via binding RAB7. Biochem Biophys Res Commun. 2015;461: 401–7. doi: 10.1016/j.bbrc.2015.04.051 25896765 10.1016/j.bbrc.2015.04.051

[19] Augenstreich J, Arbues A, Simeone R, Haanappel E, Wegener A, Sayes F, et al ESX-1 and phthiocerol dimycocerosates of Mycobacterium tuberculosis act in concert to cause phagosomal rupture and host cell apoptosis. Cell Microbiol. 2017. 10.1111/cmi.12726 28095608

[20] Tobin DM, Ramakrishnan L. Comparative pathogenesis of Mycobacterium marinum and Mycobacterium tuberculosis. Cell Microbiol. 2008;10: 1027–39. doi: 10.1111/j.1462-5822.2008.01133.x 18298637 10.1111/j.1462-5822.2008.01133.x

[21] Lerena MC, Colombo MI. Mycobacterium marinum induces a marked LC3 recruitment to its containing phagosome that depends on a functional ESX-1 secretion system. Cell Microbiol. 2011;13: 814–35. doi: 10.1111/j.1462-5822.2011.01581.x 21447143 10.1111/j.1462-5822.2011.01581.x

[22] Smith J, Manoranjan J, Pan M, Bohsali A, Xu J, Liu J, et al Evidence for pore formation in host cell membranes by ESX-1-secreted ESAT-6 and its role in Mycobacterium marinum escape from the vacuole. Infect Immun. 2008;76: 5478–87. doi: 10.1128/IAI.00614-08 18852239 10.1128/IAI.00614-08 PMC2583575

[23] Bozzaro S, Eichinger L. The professional phagocyte Dictyostelium discoideum as a model host for bacterial pathogens. Curr Drug Targets. 2011;12: 942–54. doi: 10.2174/138945011795677782 21366522 10.2174/138945011795677782 PMC3267156

[24] Mesquita A, Cardenal-Munoz E, Dominguez E, Munoz-Braceras S, Nunez-Corcuera B, Phillips BA, et al Autophagy in Dictyostelium: Mechanisms, regulation and disease in a simple biomedical model. Autophagy. 2016: 1–17. 27715405 10.1080/15548627.2016.1226737 PMC5240833

[25] Gerstenmaier L, Pilla R, Herrmann L, Herrmann H, Prado M, Villafano GJ, et al The autophagic machinery ensures nonlytic transmission of mycobacteria. Proc Natl Acad Sci U S A. 2015;112: E687–92. doi: 10.1073/pnas.1423318112 25646440 10.1073/pnas.1423318112 PMC4343083

[26] Mesquita A, Tabara LC, Martinez-Costa O, Santos-Rodrigo N, Vincent O, Escalante R. Dissecting the function of Atg1 complex in Dictyostelium autophagy reveals a connection with the pentose phosphate pathway enzyme transketolase. Open Biol. 2015;5. 10.1098/rsob.150088 PMC4554924 26246495

[27] King JS, Veltman DM, Insall RH. The induction of autophagy by mechanical stress. Autophagy. 2011;7: 1490–9. doi: 10.4161/auto.7.12.17924 22024750 10.4161/auto.7.12.17924 PMC3327616

[28] Swer PB, Lohia R, Saran S. Analysis of rapamycin induced autophagy in Dictyostelium discoideum. Indian J Exp Biol. 2014;52: 295–304. 24772931

[29] Polson HE, de Lartigue J, Rigden DJ, Reedijk M, Urbe S, Clague MJ, et al Mammalian Atg18 (WIPI2) localizes to omegasome-anchored phagophores and positively regulates LC3 lipidation. Autophagy. 2010;6: 506–22. doi: 10.4161/auto.6.4.11863 20505359 10.4161/auto.6.4.11863

[30] Klionsky DJ, Abdelmohsen K, Abe A, Abedin MJ, Abeliovich H, Acevedo Arozena A, et al Guidelines for the use and interpretation of assays for monitoring autophagy (3rd edition). Autophagy. 2016;12: 1–222. doi: 10.1080/15548627.2015.1100356 26799652 10.1080/15548627.2015.1100356 PMC4835977

[31] Gao M, Yeh PY, Lu YS, Hsu CH, Chen KF, Lee WC, et al OSU-03012, a novel celecoxib derivative, induces reactive oxygen species-related autophagy in hepatocellular carcinoma. Cancer Res. 2008;68: 9348–57. doi: 10.1158/0008-5472.CAN-08-1642 19010909 10.1158/0008-5472.CAN-08-1642

[32] Huang S, Yang ZJ, Yu C, Sinicrope FA. Inhibition of mTOR kinase by AZD8055 can antagonize chemotherapy-induced cell death through autophagy induction and down-regulation of p62/sequestosome 1. J Biol Chem. 2011;286: 40002–12. doi: 10.1074/jbc.M111.297432 21949121 10.1074/jbc.M111.297432 PMC3220585

[33] Fan QW, Knight ZA, Goldenberg DD, Yu W, Mostov KE, Stokoe D, et al A dual PI3 kinase/mTOR inhibitor reveals emergent efficacy in glioma. Cancer Cell. 2006;9: 341–9. doi: 10.1016/j.ccr.2006.03.029 16697955 10.1016/j.ccr.2006.03.029 PMC2925230

[34] Otto GP, Wu MY, Kazgan N, Anderson OR, Kessin RH. Dictyostelium macroautophagy mutants vary in the severity of their developmental defects. J Biol Chem. 2004;279: 15621–9. doi: 10.1074/jbc.M311139200 14736886 10.1074/jbc.M311139200

[35] Hagedorn M, Soldati T. Flotillin and RacH modulate the intracellular immunity of Dictyostelium to Mycobacterium marinum infection. Cell Microbiol. 2007;9: 2716–33. doi: 10.1111/j.1462-5822.2007.00993.x 17587329 10.1111/j.1462-5822.2007.00993.x

[36] Gutierrez MG, Vazquez CL, Munafo DB, Zoppino FC, Beron W, Rabinovitch M, et al Autophagy induction favours the generation and maturation of the Coxiella-replicative vacuoles. Cell Microbiol. 2005;7: 981–93. doi: 10.1111/j.1462-5822.2005.00527.x 15953030 10.1111/j.1462-5822.2005.00527.x

[37] Pujol C, Klein KA, Romanov GA, Palmer LE, Cirota C, Zhao Z, et al Yersinia pestis can reside in autophagosomes and avoid xenophagy in murine macrophages by preventing vacuole acidification. Infect Immun. 2009;77: 2251–61. doi: 10.1128/IAI.00068-09 19289509 10.1128/IAI.00068-09 PMC2687347

[38] Collins CA, De Maziere A, van Dijk S, Carlsson F, Klumperman J, Brown EJ. Atg5-independent sequestration of ubiquitinated mycobacteria. PLoS Pathog. 2009;5: e1000430 doi: 10.1371/journal.ppat.1000430 19436699 10.1371/journal.ppat.1000430 PMC2673685

[39] Peng X, Jiang G, Liu W, Zhang Q, Qian W, Sun J. Characterization of differential pore-forming activities of ESAT-6 proteins from Mycobacterium tuberculosis and Mycobacterium smegmatis. FEBS Lett. 2016;590: 509–19. doi: 10.1002/1873-3468.12072 26801203 10.1002/1873-3468.12072 PMC4973571

[40] Abdel-Nour M, Tsalikis J, Kleinman D, Girardin SE. The emerging role of mTOR signalling in antibacterial immunity. Immunol Cell Biol. 2014;92: 346–53. doi: 10.1038/icb.2014.3 24518980 10.1038/icb.2014.3

[41] Gao LY, Guo S, McLaughlin B, Morisaki H, Engel JN, Brown EJ. A mycobacterial virulence gene cluster extending RD1 is required for cytolysis, bacterial spreading and ESAT-6 secretion. Mol Microbiol. 2004;53: 1677–93. doi: 10.1111/j.1365-2958.2004.04261.x 15341647 10.1111/j.1365-2958.2004.04261.x

[42] Pym AS, Brodin P, Brosch R, Huerre M, Cole ST. Loss of RD1 contributed to the attenuation of the live tuberculosis vaccines Mycobacterium bovis BCG and Mycobacterium microti. Mol Microbiol. 2002;46: 709–17. 12410828 10.1046/j.1365-2958.2002.03237.x

[43] Lamb CA, Yoshimori T, Tooze SA. The autophagosome: origins unknown, biogenesis complex. Nat Rev Mol Cell Biol. 2013;14: 759–74. doi: 10.1038/nrm3696 24201109 10.1038/nrm3696

[44] Yu L, McPhee CK, Zheng L, Mardones GA, Rong Y, Peng J, et al Termination of autophagy and reformation of lysosomes regulated by mTOR. Nature. 2010;465: 942–6. doi: 10.1038/nature09076 20526321 10.1038/nature09076 PMC2920749

[45] Zhang J, Zhou W, Lin J, Wei P, Zhang Y, Jin P, et al Autophagic lysosomal reformation depends on mTOR reactivation in H2O2-induced autophagy. Int J Biochem Cell Biol. 2016;70: 76–81. doi: 10.1016/j.biocel.2015.11.009 26589722 10.1016/j.biocel.2015.11.009

[46] Sancak Y, Bar-Peled L, Zoncu R, Markhard AL, Nada S, Sabatini DM. Ragulator-Rag complex targets mTORC1 to the lysosomal surface and is necessary for its activation by amino acids. Cell. 2010;141: 290–303. doi: 10.1016/j.cell.2010.02.024 20381137 10.1016/j.cell.2010.02.024 PMC3024592

[47] Binda M, Peli-Gulli MP, Bonfils G, Panchaud N, Urban J, Sturgill TW, et al The Vam6 GEF controls TORC1 by activating the EGO complex. Mol Cell. 2009;35: 563–73. doi: 10.1016/j.molcel.2009.06.033 19748353 10.1016/j.molcel.2009.06.033

[48] Bar-Peled L, Sabatini DM. Regulation of mTORC1 by amino acids. Trends Cell Biol. 2014;24: 400–6. doi: 10.1016/j.tcb.2014.03.003 24698685 10.1016/j.tcb.2014.03.003 PMC4074565

[49] Steinert M. Pathogen-host interactions in Dictyostelium, Legionella, Mycobacterium and other pathogens. Semin Cell Dev Biol. 2011;22: 70–6. doi: 10.1016/j.semcdb.2010.11.003 21109012 10.1016/j.semcdb.2010.11.003

[50] van der Vaart M, Korbee CJ, Lamers GE, Tengeler AC, Hosseini R, Haks MC, et al The DNA damage-regulated autophagy modulator DRAM1 links mycobacterial recognition via TLR-MYD88 to autophagic defense [corrected]. Cell Host Microbe. 2014;15: 753–67. doi: 10.1016/j.chom.2014.05.005 24922577 10.1016/j.chom.2014.05.005

[51] Mizushima N. The role of the Atg1/ULK1 complex in autophagy regulation. Curr Opin Cell Biol. 2010;22: 132–9. doi: 10.1016/j.ceb.2009.12.004 20056399 10.1016/j.ceb.2009.12.004

[52] Geng J, Klionsky DJ. The Atg8 and Atg12 ubiquitin-like conjugation systems in macroautophagy. 'Protein modifications: beyond the usual suspects' review series. EMBO Rep. 2008;9: 859–64. doi: 10.1038/embor.2008.163 18704115 10.1038/embor.2008.163 PMC2529362

[53] Kimmey JM, Huynh JP, Weiss LA, Park S, Kambal A, Debnath J, et al Unique role for ATG5 in neutrophil-mediated immunopathology during M. tuberculosis infection. Nature. 2015;528: 565–9. doi: 10.1038/nature16451 26649827 10.1038/nature16451 PMC4842313

[54] Kreibich S, Emmenlauer M, Fredlund J, Ramo P, Munz C, Dehio C, et al Autophagy Proteins Promote Repair of Endosomal Membranes Damaged by the Salmonella Type Three Secretion System 1. Cell Host Microbe. 2015;18: 527–37. doi: 10.1016/j.chom.2015.10.015 26567507 10.1016/j.chom.2015.10.015

[55] Watson RO, Manzanillo PS, Cox JS. Extracellular M. tuberculosis DNA targets bacteria for autophagy by activating the host DNA-sensing pathway. Cell. 2012;150: 803–15. doi: 10.1016/j.cell.2012.06.040 22901810 10.1016/j.cell.2012.06.040 PMC3708656

[56] Singh SB, Davis AS, Taylor GA, Deretic V. Human IRGM induces autophagy to eliminate intracellular mycobacteria. Science. 2006;313: 1438–41. doi: 10.1126/science.1129577 16888103 10.1126/science.1129577

[57] Shaughnessy LM, Hoppe AD, Christensen KA, Swanson JA. Membrane perforations inhibit lysosome fusion by altering pH and calcium in Listeria monocytogenes vacuoles. Cell Microbiol. 2006;8: 781–92. doi: 10.1111/j.1462-5822.2005.00665.x 16611227 10.1111/j.1462-5822.2005.00665.x PMC1435990

[58] Malet JK, Cossart P, Ribet D. Alteration of epithelial cell lysosomal integrity induced by bacterial cholesterol-dependent cytolysins. Cell Microbiol. 2017;19. 10.1111/cmi.12682 PMC5347955 27739224

[59] Zullo AJ, Lee S. Mycobacterial induction of autophagy varies by species and occurs independently of mammalian target of rapamycin inhibition. J Biol Chem. 2012;287: 12668–78. doi: 10.1074/jbc.M111.320135 22275355 10.1074/jbc.M111.320135 PMC3339952

[60] Dong H, Jing W, Runpeng Z, Xuewei X, Min M, Ru C, et al ESAT6 inhibits autophagy flux and promotes BCG proliferation through MTOR. Biochem Biophys Res Commun. 2016. 10.1016/j.bbrc.2016.06.042 27317487

[61] Faix J, Kreppel L, Shaulsky G, Schleicher M, Kimmel AR. A rapid and efficient method to generate multiple gene disruptions in Dictyostelium discoideum using a single selectable marker and the Cre-loxP system. Nucleic Acids Res. 2004;32: e143 doi: 10.1093/nar/gnh136 15507682 10.1093/nar/gnh136 PMC528815

[62] Arafah S, Kicka S, Trofimov V, Hagedorn M, Andreu N, Wiles S, et al Setting up and monitoring an infection of Dictyostelium discoideum with mycobacteria. Methods Mol Biol. 2013;983: 403–17. doi: 10.1007/978-1-62703-302-2_22 23494320 10.1007/978-1-62703-302-2_22

[63] Foster KG, Acosta-Jaquez HA, Romeo Y, Ekim B, Soliman GA, Carriere A, et al Regulation of mTOR complex 1 (mTORC1) by raptor Ser863 and multisite phosphorylation. J Biol Chem. 2010;285: 80–94. doi: 10.1074/jbc.M109.029637 19864431 10.1074/jbc.M109.029637 PMC2804229

[64] Gingras AC, Gygi SP, Raught B, Polakiewicz RD, Abraham RT, Hoekstra MF, et al Regulation of 4E-BP1 phosphorylation: a novel two-step mechanism. Genes Dev. 1999;13: 1422–37. 10364159 10.1101/gad.13.11.1422 PMC316780

[65] Ravanel K, de Chassey B, Cornillon S, Benghezal M, Zulianello L, Gebbie L, et al Membrane sorting in the endocytic and phagocytic pathway of Dictyostelium discoideum. Eur J Cell Biol. 2001;80: 754–64. doi: 10.1078/0171-9335-00215 11831389 10.1078/0171-9335-00215

[66] Dieckmann R, von Heyden Y, Kistler C, Gopaldass N, Hausherr S, Crawley SW, et al A myosin IK-Abp1-PakB circuit acts as a switch to regulate phagocytosis efficiency. Mol Biol Cell. 2010;21: 1505–18. doi: 10.1091/mbc.E09-06-0485 20200225 10.1091/mbc.E09-06-0485 PMC2861610

[67] Laemmli UK. Cleavage of structural proteins during the assembly of the head of bacteriophage T4. Nature. 1970;227: 680–5. 5432063 10.1038/227680a0

[68] Schwarz EC, Neuhaus EM, Kistler C, Henkel AW, Soldati T. Dictyostelium myosin IK is involved in the maintenance of cortical tension and affects motility and phagocytosis. J Cell Sci. 2000;113 (Pt 4): 621–33. 10652255 10.1242/jcs.113.4.621

[69] Hagedorn M, Neuhaus EM, Soldati T. Optimized fixation and immunofluorescence staining methods for Dictyostelium cells. Methods Mol Biol. 2006;346: 327–38. doi: 10.1385/1-59745-144-4:327 16957300 10.1385/1-59745-144-4:327

[70] Barisch C, Lopez-Jimenez AT, Soldati T. Live imaging of Mycobacterium marinum infection in Dictyostelium discoideum. Methods Mol Biol. 2015;1285: 369–85. doi: 10.1007/978-1-4939-2450-9_23 25779329 10.1007/978-1-4939-2450-9_23

[71] Marchetti A, Mercanti V, Cornillon S, Alibaud L, Charette SJ, Cosson P. Formation of multivesicular endosomes in Dictyostelium. J Cell Sci. 2004;117: 6053–9. doi: 10.1242/jcs.01524 15536120 10.1242/jcs.01524

[72] Barisch C, Paschke P, Hagedorn M, Maniak M, Soldati T. Lipid droplet dynamics at early stages of Mycobacterium marinum infection in Dictyostelium. Cell Microbiol. 2015;17: 1332–49. doi: 10.1111/cmi.12437 25772333 10.1111/cmi.12437

[73] Calvo-Garrido J, Carilla-Latorre S, Mesquita A, Escalante R. A proteolytic cleavage assay to monitor autophagy in Dictyostelium discoideum. Autophagy. 2011;7: 1063–8. doi: 10.4161/auto.7.9.16629 21876387 10.4161/auto.7.9.16629

[74] Phipson B, Smyth GK. Permutation P-values should never be zero: calculating exact P-values when permutations are randomly drawn. Stat Appl Genet Mol Biol. 2010;9: Article39. 10.2202/1544-6115.1585 21044043

[75] Tattoli I, Sorbara MT, Yang C, Tooze SA, Philpott DJ, Girardin SE. Listeria phospholipases subvert host autophagic defenses by stalling pre-autophagosomal structures. EMBO J. 2013;32: 3066–78. doi: 10.1038/emboj.2013.234 24162724 10.1038/emboj.2013.234 PMC3844955

[76] Tattoli I, Philpott DJ, Girardin SE. The bacterial and cellular determinants controlling the recruitment of mTOR to the Salmonella-containing vacuole. Biol Open. 2012;1: 1215–25. doi: 10.1242/bio.20122840 23259056 10.1242/bio.20122840 PMC3522883

[77] Tattoli I, Sorbara MT, Vuckovic D, Ling A, Soares F, Carneiro LA, et al Amino acid starvation induced by invasive bacterial pathogens triggers an innate host defense program. Cell Host Microbe. 2012;11: 563–75. doi: 10.1016/j.chom.2012.04.012 22704617 10.1016/j.chom.2012.04.012

