# Supplementary material for: Mycobacterium marinum antagonistically induces an autophagic response while repressing the autophagic flux in a TORC1- and ESX-1-dependent manner
Source: PLoS Pathog. 2017 Apr 17;13(4):e1006344. doi: 10.1371/journal.ppat.1006344 (PMC5407849; doi:10.1371/journal.ppat.1006344)
Supplement: S1 References — (DOCX) [file ppat.1006344.s001.docx]

**Supporting References**

1. King JS, Gueho A, Hagedorn M, Gopaldass N, Leuba F, Soldati T, et al. WASH is required for lysosomal recycling and efficient autophagic and phagocytic digestion. Mol Biol Cell. 2013;24: 2714-26.

2. Veltman DM, Akar G, Bosgraaf L, Van Haastert PJ. A new set of small, extrachromosomal expression vectors for Dictyostelium discoideum. Plasmid. 2009;61: 110-8.

3. Dieckmann R, von Heyden Y, Kistler C, Gopaldass N, Hausherr S, Crawley SW, et al. A myosin IK-Abp1-PakB circuit acts as a switch to regulate phagocytosis efficiency. Mol Biol Cell. 2010;21: 1505-18.

4. King JS, Veltman DM, Insall RH. The induction of autophagy by mechanical stress. Autophagy. 2011;7: 1490-9.

5. Barisch C, Lopez-Jimenez AT, Soldati T. Live imaging of Mycobacterium marinum infection in Dictyostelium discoideum. Methods Mol Biol. 2015;1285: 369-85.

6. Carnell M, Zech T, Calaminus SD, Ura S, Hagedorn M, Johnston SA, et al. Actin polymerization driven by WASH causes V-ATPase retrieval and vesicle neutralization before exocytosis. J Cell Biol. 2011;193: 831-9.

7. Carroll P, Schreuder LJ, Muwanguzi-Karugaba J, Wiles S, Robertson BD, Ripoll J, et al. Sensitive detection of gene expression in mycobacteria under replicating and non-replicating conditions using optimized far-red reporters. PLoS One. 2010;5: e9823.

8. Cosma CL, Humbert O, Ramakrishnan L. Superinfecting mycobacteria home to established tuberculous granulomas. Nat Immunol. 2004;5: 828-35.

9. Cosma CL, Humbert O, Sherman DR, Ramakrishnan L. Trafficking of superinfecting Mycobacterium organisms into established granulomas occurs in mammals and is independent of the Erp and ESX-1 mycobacterial virulence loci. J Infect Dis. 2008;198: 1851-5.

10. Andreu N, Zelmer A, Fletcher T, Elkington PT, Ward TH, Ripoll J, et al. Optimisation of bioluminescent reporters for use with mycobacteria. PLoS One. 2010;5: e10777.

11. Pym AS, Brodin P, Brosch R, Huerre M, Cole ST. Loss of RD1 contributed to the attenuation of the live tuberculosis vaccines Mycobacterium bovis BCG and Mycobacterium microti. Mol Microbiol. 2002;46: 709-17.
