# Supplementary figures and images for: Mycobacterium marinum antagonistically induces an autophagic response while repressing the autophagic flux in a TORC1- and ESX-1-dependent manner
Source: PLoS Pathog. 2017 Apr 17;13(4):e1006344. doi: 10.1371/journal.ppat.1006344 (PMC5407849; doi:10.1371/journal.ppat.1006344)

**S1 Fig**

**A**

GFP-Atg8

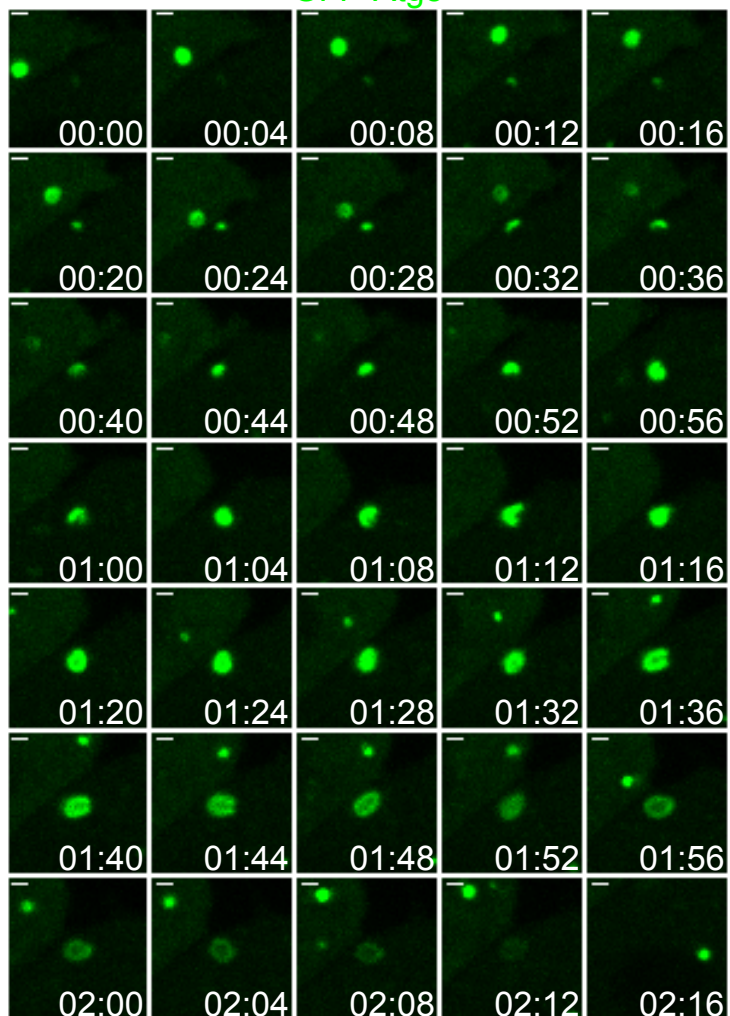

**B**

GFP-Atg8

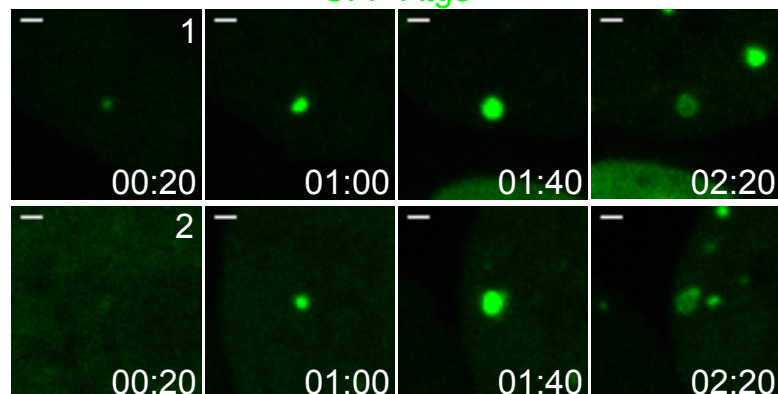

**C**

GFP-Atg8

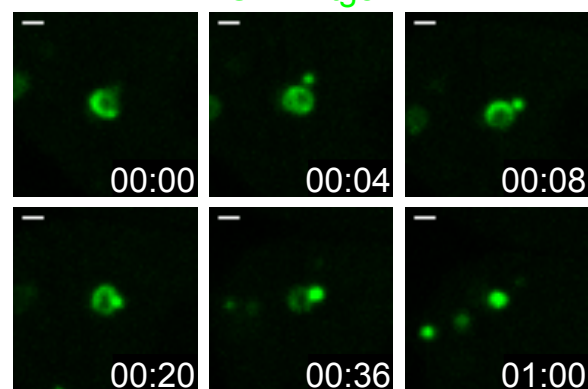

**D**

GFP-Atg18 *Mm*

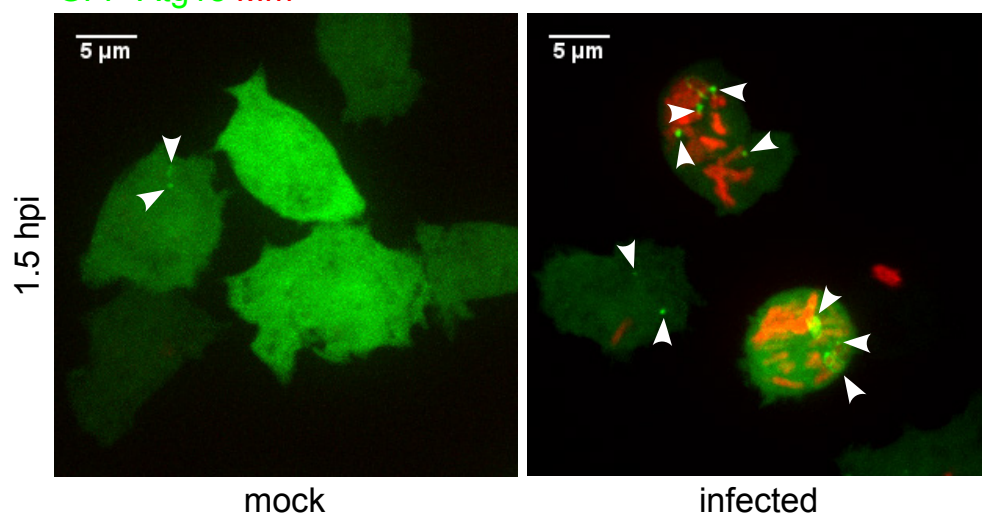

**E**

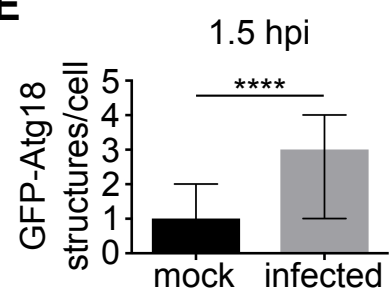

Supplement: S1 Fig — A. Video frames showing the formation and degradation of one GFP-Atg8+ autophagosome. Scale bars, 1 μm; B. Video frames of two more examples of GFP-Atg8+ autophagosomes flux in D. discoideum. Scale bars, 1 μm; C. Time-lapse captures of one fusion event among GFP-Atg8+ vesicles in D. discoideum. Scale bars, 1 μm; D. Two representative maximum projections of GFP-Atg18-expressing D. discoideum cells infected (right) or not (left) with mCherry-expressing M. marinum wt. Images were recorded live 1.5 h after infection. White arrowheads point to GFP-Atg18 structures. Scale bars, 5 μm; E. Median and interquartile ranges of the number of GFP-Atg18 structures per cell at 1.5 hpi. 57–97 cells from two independent experiments were counted. Unpaired t test (****p ≤ 0.0001). (PDF) [file ppat.1006344.s002.pdf]

**S2 Fig****A**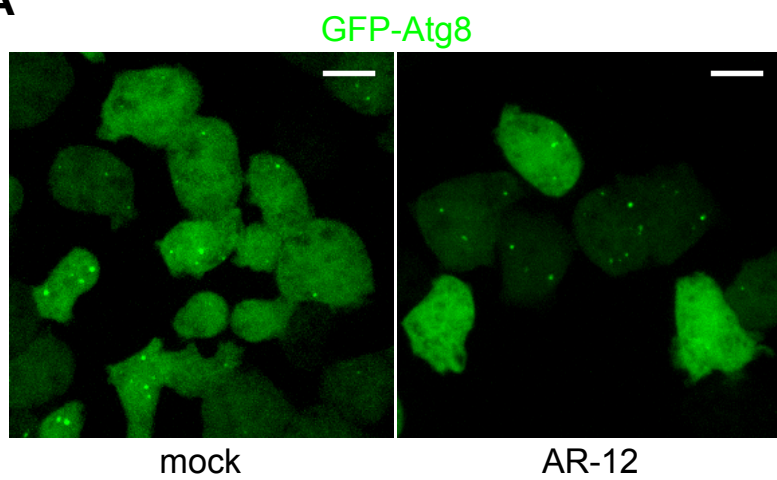**B**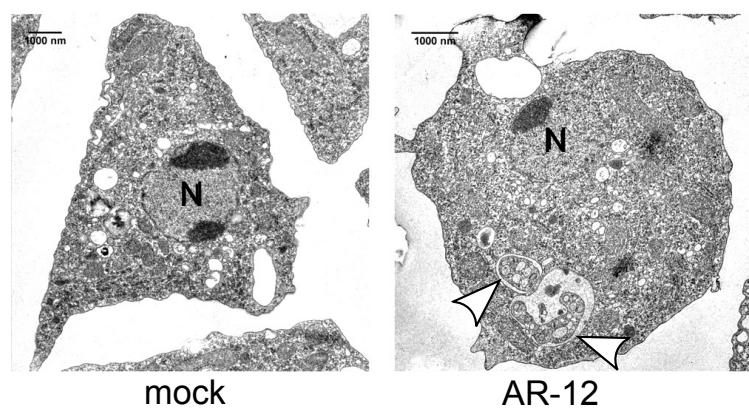**C**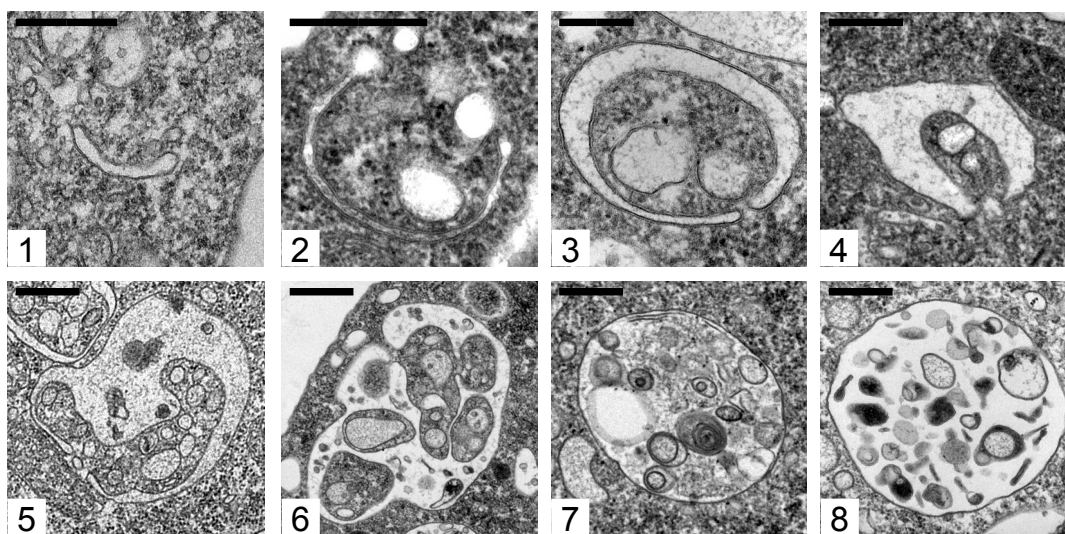**D**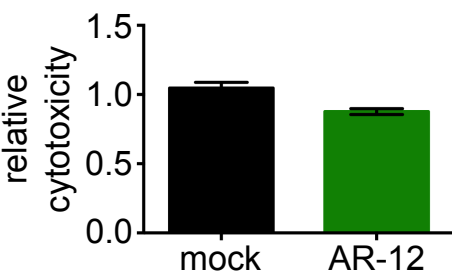**E**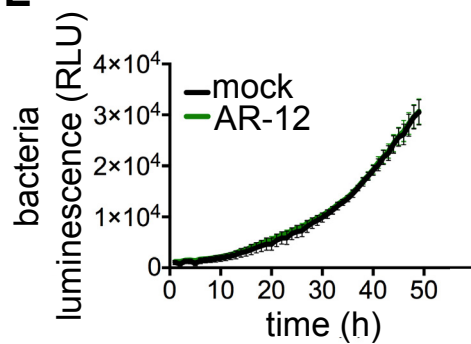**F**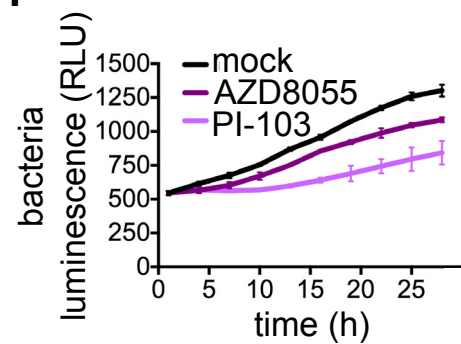

Supplement: S2 Fig — A. Representative maximum projections of live GFP-Atg8-expressing D. discoideum treated or not with AR-12 at 2.5 μM for 2 hours. Scale bars, 10 μm; B. EM of GFP-Atg8-expressing D. discoideum cells treated or not with AR-12 at 5 μM for 1 hour. Nuclei are labelled by the letter . White arrowheads label large double membrane compartments engulfing cytosolic material. Scale bars, 1 μm; C. Reconstruction by EM of the autophagosome formation events in D. discoideum after one hour incubation with AR-12 at 5 μM: 1. nucleation; 2–6. elongation; 7. closure; 8. maturation. Scale bars, 0.4 μm; D. Cell cytotoxicity of D. discoideum treated or not with AR-12 at 2.5 μM for 4 h. Mean and standard deviation of three experiments; E. AR-12 at 2.5 μM was added or not to M. marinum and the bacterial growth was monitored (RLU) in triplicates; F. Cells infected with lux-expressing M. marinum wt bacteria were treated or not with AZD8055 or PI-103 at 2.5 μM. Intracellular bacterial growth (RLU) is represented as the mean and standard deviation from duplicates. (PDF) [file ppat.1006344.s003.pdf]

S3 Fig

A

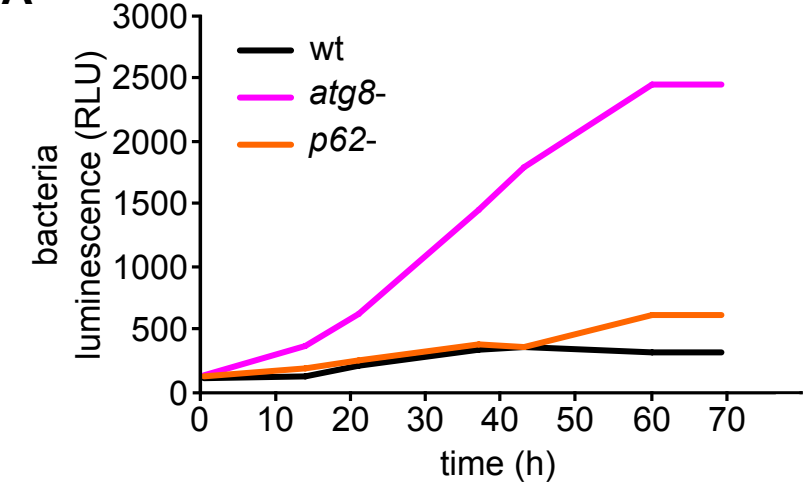

B

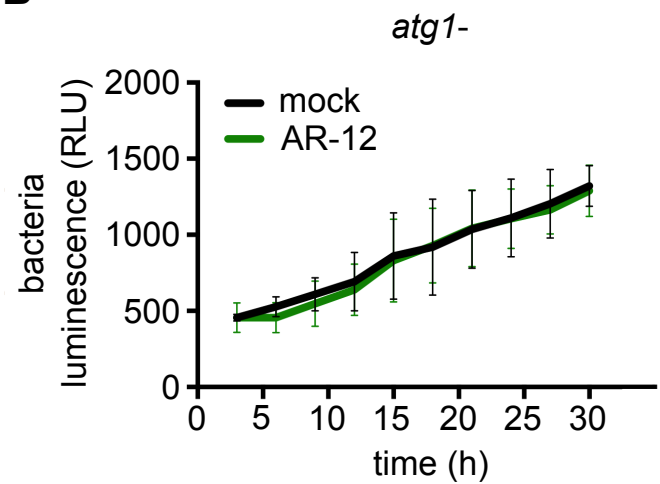

Supplement: S3 Fig — A. D. discoideum wt, atg8- and p62- cells were infected with lux-expressing M. marinum wt and intracellular bacteria growth was measured (RLU). One representative experiment of three; B. D. discoideum atg1- cells were infected with lux-expressing M. marinum wt and treated or not with AR-12 at 2.5 μM. The intracellular bacterial growth was monitored as RLUs. The average of the RLUs from three consecutive time points, in triplicates, is represented. (PDF) [file ppat.1006344.s004.pdf]

S4 Fig

A

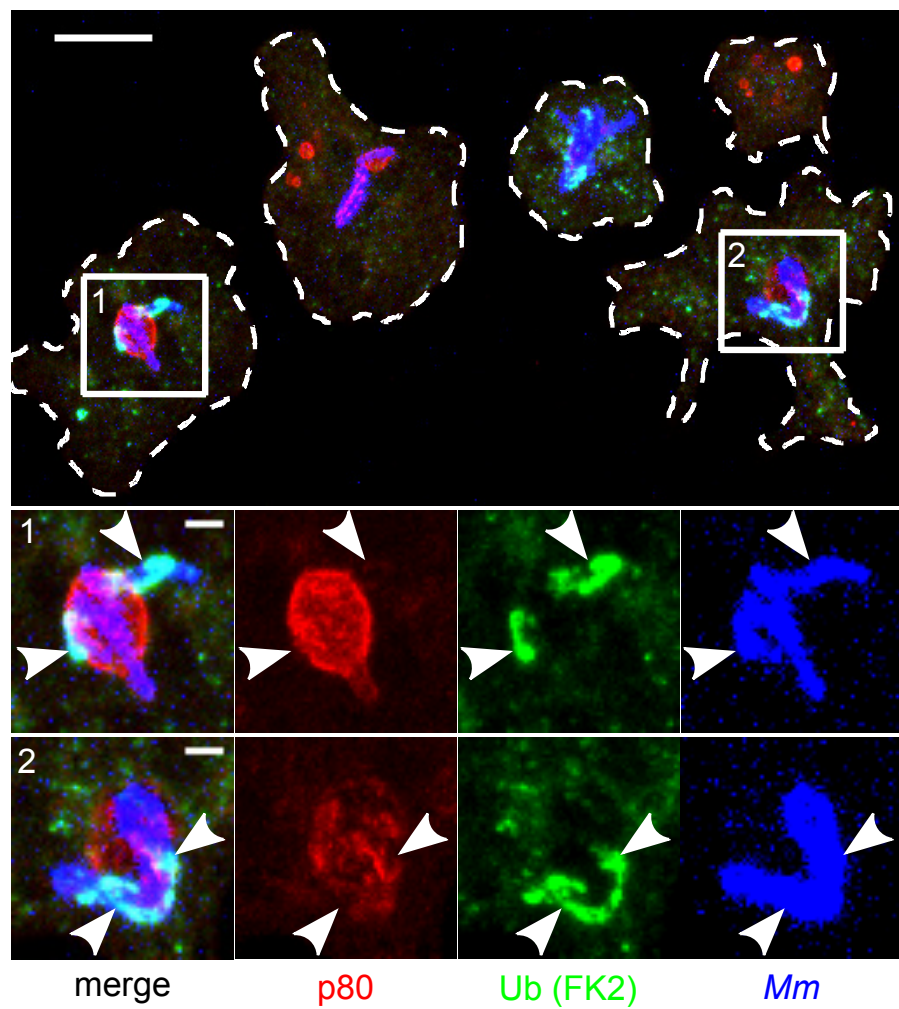

B

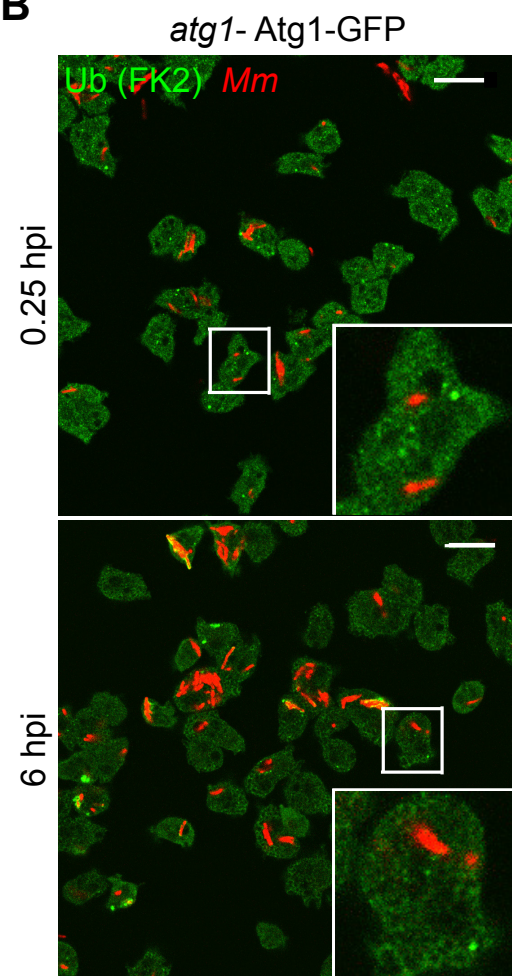

C

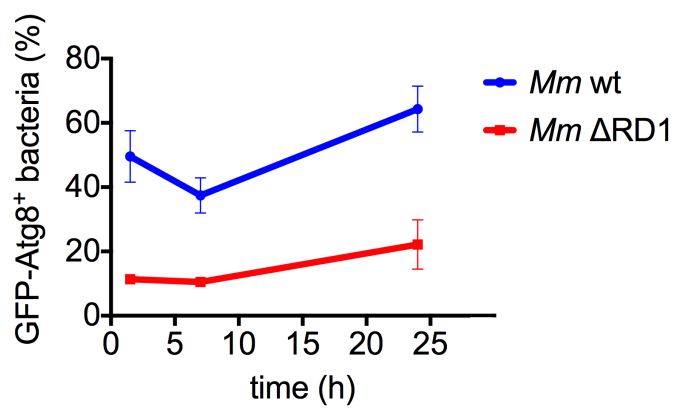

D

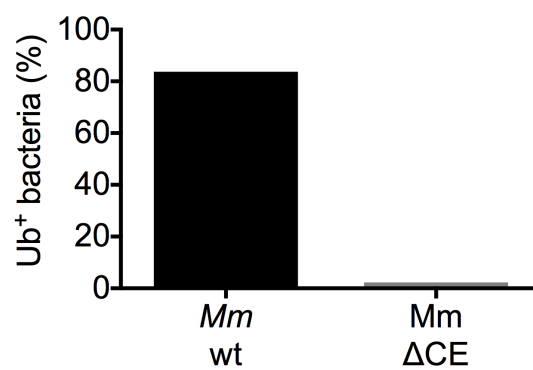

Supplement: S4 Fig — A. D. discoideum was infected with mCherry-expressing M. marinum, fixed and stained against p80 (red), Ub (green) and mCherry (blue). Representative maximum projections at 24 hpi. White arrowheads point to ubiquitination. Scale bars, 10 μm (upper panel) and 2 μm (panels 1 and 2); B. D. discoideum atg1- Atg1-GFP cells were infected with mCherry-expressing M. marinum, fixed and stained for Ub (green) and mCherry (red). Representative maximum projections at 0.25 and 6 hpi. Scale bars, 10 μm. C. Infections represented in Fig 4A were also quantified as the percentage of M. marinum wt and ΔRD1 (expressing mCherry and DsRed, respectively) positive for GFP-Atg8 at 1.5, 7 and 24 hpi. Means and standard deviations from independent triplicates. A mean of 171 and 93 MCVs per time point was counted for M. marinum wt and ΔRD1 infection, respectively; D. D. discoideum atg1- was infected with M. marinum wt and ΔCE (both expressing mCherry), fixed and stained for Ub (green) and mCherry (red). Quantification of the percentage of bacteria (red) positive for Ub (green) at 6 hpi. (PDF) [file ppat.1006344.s005.pdf]

**S5 Fig**

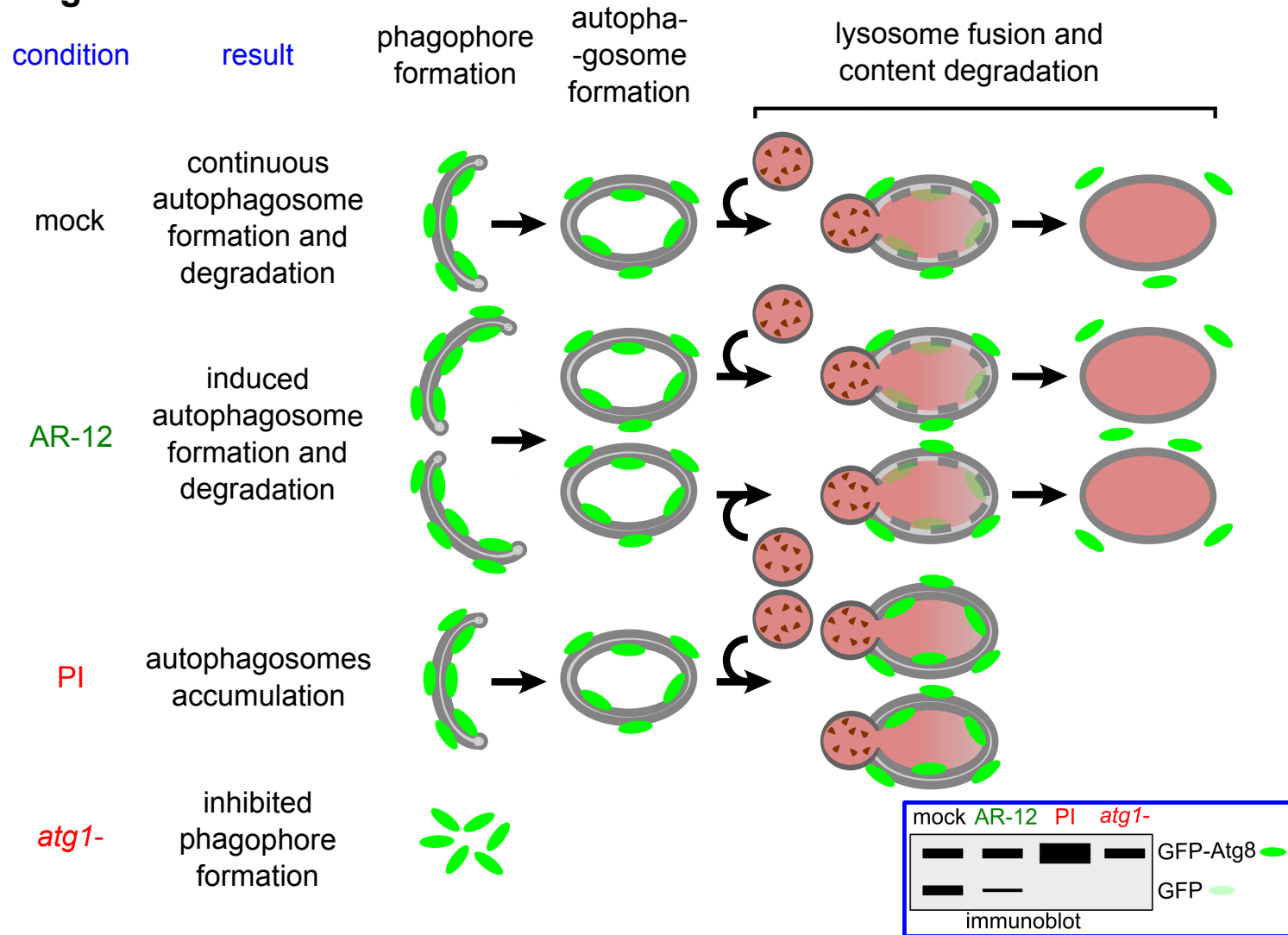

Supplement: S5 Fig — Scheme of the autophagic flux assay in D. discoideum expressing exogenous GFP-Atg8 and the expected results. In mock conditions autophagosomes form and fusion with lysosomes, leading to the degradation of the engulfed cytoplasmic material and the GFP-Atg8 protein at the inner membranes. Atg8 is sensitive to degradation whereas GFP, which fluorescence is quenched by low pH, is relatively resistant to hydrolysis [30]. Therefore, after autolysosome formation low signal is detected by fluorescence microscopy while free GFP appears in immunoblotting; Treatment with AR-12 induces both autophagosomes formation and autophagic degradation, while the rates of GFP-Atg8 production in the cell remains the same. As a result, more autophagosomes can be observed by microscopy while the intensity of the free GFP band in immunoblotting is reduced; Short-treatment (1 h) with PI derives in the accumulation of autophagosomes which content cannot be degraded. Hence, by both microscopy and immunoblot higher GFP-Atg8 signal will be observed. Double treatment with AR-12 and PI arises the accumulation of autophagosomes; In atg1- cells, autophagosomes cannot be formed under any condition. As a consequence, the GFP-Atg8 signal does not vary during the experiment. It needs to be noticed that some Atg1-independent degradation of GFP-Atg8 may still occur. (PDF) [file ppat.1006344.s006.pdf]

**S6 Fig**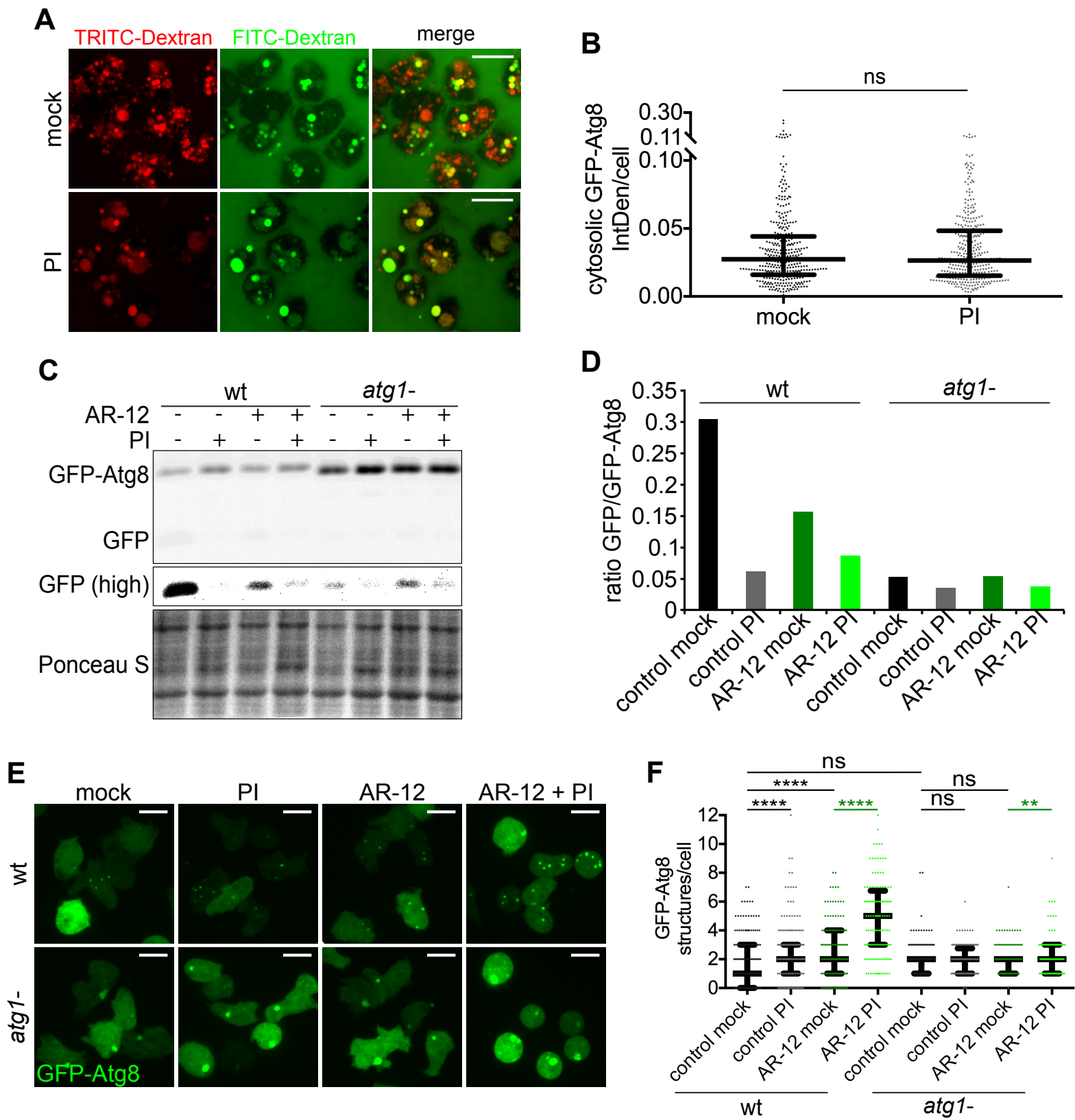

Supplement: S6 Fig — A. D. discoideum cells were incubated over night with TRITC-Dextran (pH-insensitive probe that labels the endo-lysosomal pathway in red) and FITC-Dextran (green pH-sensitive probe rapidly bleached upon acidification) followed or not by one hour of treatment with PI at 2.5×. Shown are representative maximum projections of live cells. Increase in the number and/or size of yellow vesicles after PI treatment indicates decrease in the number of acidic compartments. Scale bars, 10 μm; B. GFP-Atg8-expressing cells were incubated or not with PI at 2.5× for one hour. Maximum projections were used to measure the IntDen of the cytosolic GFP-Atg8 fluorescence compared to the extracellular IntDen (background). Median with interquartile ranges of the cytosolic GFP-Atg8 IntDen per cell. Each dot represents one cell. More than 350 cells per condition from three independent experiments were counted. Mann-Whitney test (ns, p > 0.05); C. GFP-Atg8-expressing D. discoideum wt or atg1- cells were treated or not with AR-12 at 2.5 μm. One hour before the end of the treatment, cells were incubated or not with PI at 2.5× and immunoblotted against GFP. Ponceau-S staining was used as loading control; D. Ratio GFP/GFP-Atg8 from the immunoblot represented in C.; E. Representative maximum projections of live GFP-Atg8-expressing D. discoideum wt and atg1- cells under the treatments described in C. Scale bars, 10 μm; F. Median with interquartile ranges of the number of GFP-Atg8 structures per cell during the treatments carried out in C. and E. Each dot represents one cell. 116–178 cells per condition were counted. The values of λ that define the Poisson distribution of each data set and differences between them were calculated as described in Materials and Methods (**p ≤ 0.01; ****p ≤ 0.0001; ns, p > 0.05). (PDF) [file ppat.1006344.s007.pdf]

**S7 Fig**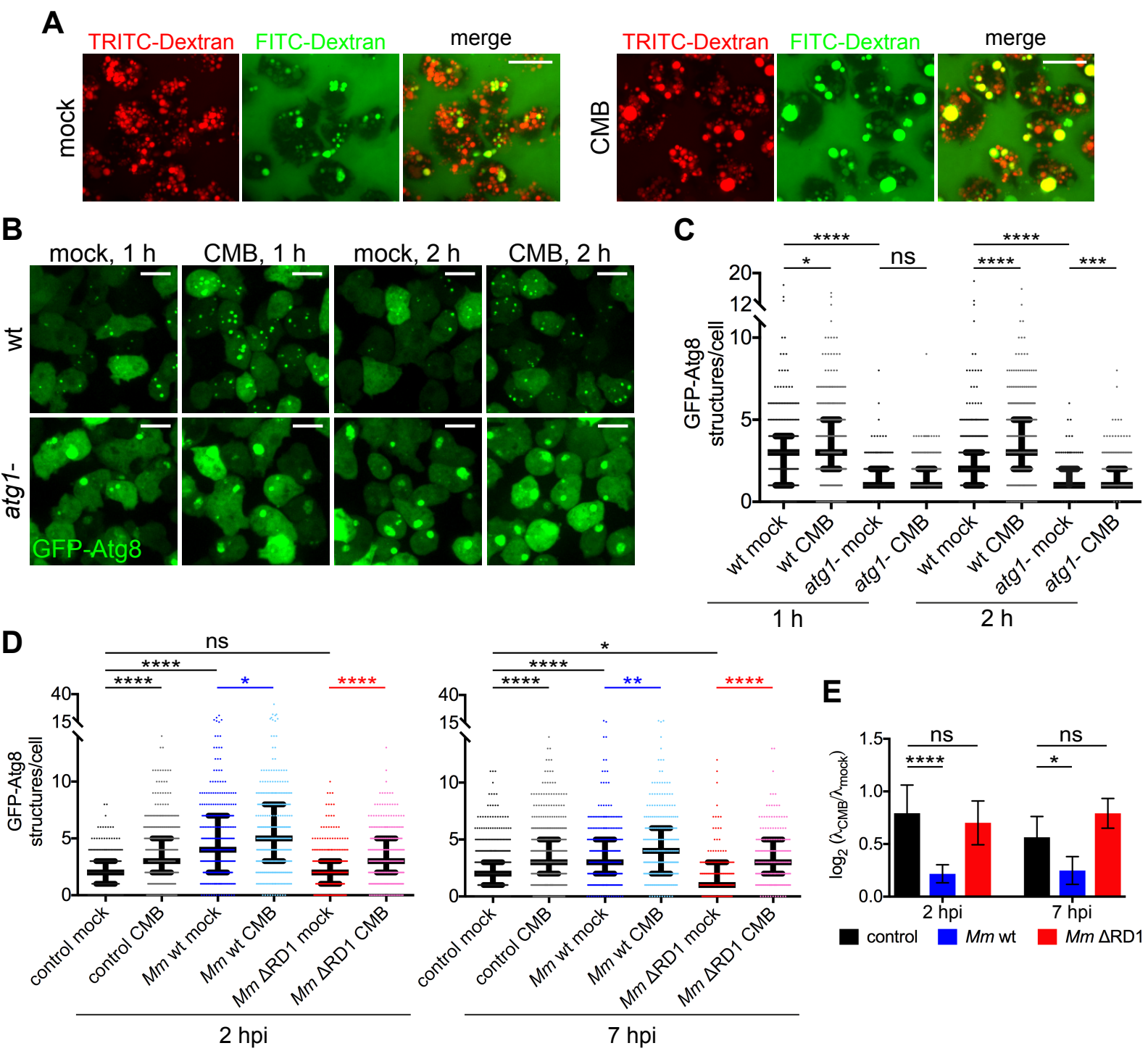

Supplement: S7 Fig — A. D. discoideum cells were incubated over night with TRITC- and FITC-Dextran followed by 2 h of treatment with 1 μM CMB (DMSO as mock). Shown are representative maximum projections of live cells. Increase in the number and/or size of yellow vesicles after CMB treatment indicates decrease in the number of acidic compartments. Scale bars, 10 μm; B. Representative maximum projections of live GFP-Atg8-expressing D. discoideum wt and atg1- cells treated or mock-treated with CMB for 1 or 2 h. Scale bars, 10 μm; C. Median with interquartile ranges of the number of GFP-Atg8 structures per cell during the treatments carried out in B. Each dot represents one cell. 237–439 cells per condition were counted. The values of λ that define the Poisson distribution of each data set and differences between them were calculated as described in Materials and Methods (*p ≤ 0.05; ***p ≤ 0.001; ****p ≤ 0.0001; ns, p > 0.05); D. GFP-Atg8-expressing cells were infected or mock-infected for 0.5 or 5.5 h with mCherry-expressing M. marinum wt or with DsRed-expressing M. marinum ΔRD1 and treated or not with 1 μM CMB for 1.5 additional hours. Medians with interquartile ranges of the number of GFP-Atg8 structures per cell. Each dot represents one cell. 164–551 cells per condition from three independent experiments were counted. The values of λ that define the Poisson distribution of each data set and differences between them were calculated as described in Materials and Methods (*p ≤ 0.05; **p ≤ 0.01; ****p ≤ 0.0001; ns, p > 0.05); E. Mean and standard deviation of the log2 (λCMB/λmock) from the three independent replicates represented in D. A log2 (λCMB/λmock) of zero implies that there was a total autophagic block. p-values calculated as described in Materials and Methods (*p ≤ 0.05; (****p ≤ 0.0001; ns, p > 0.05). (PDF) [file ppat.1006344.s008.pdf]

# S8 Fig

**A**

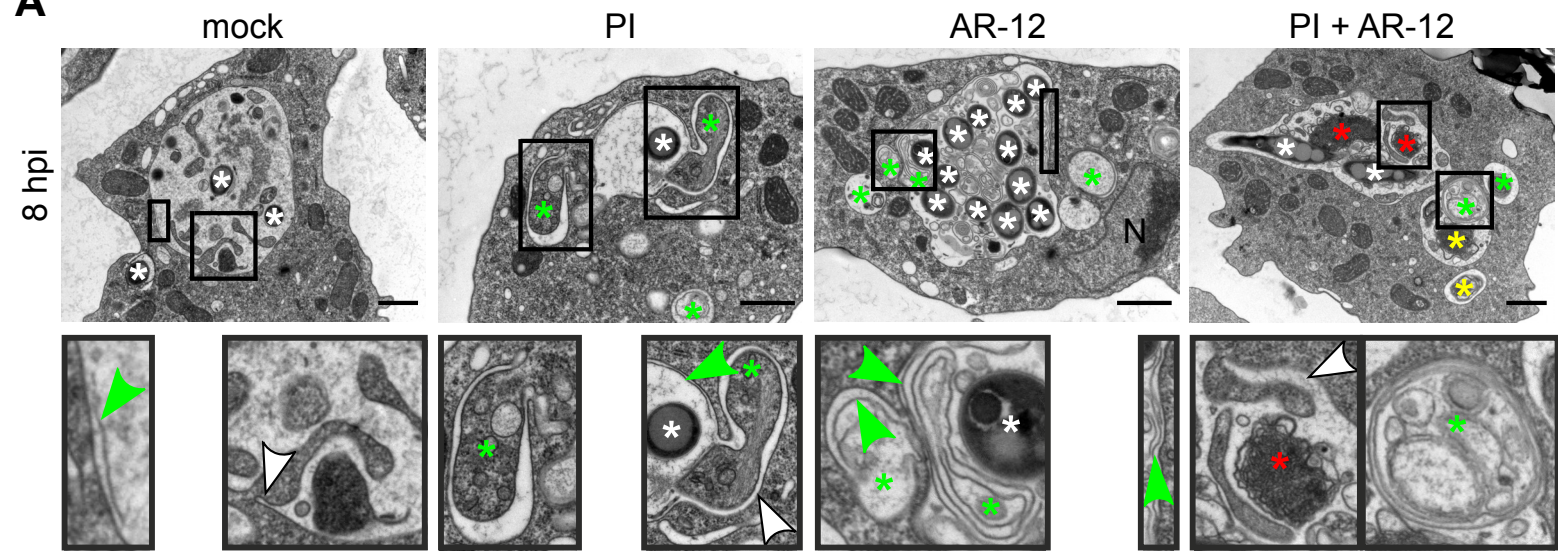

**B**

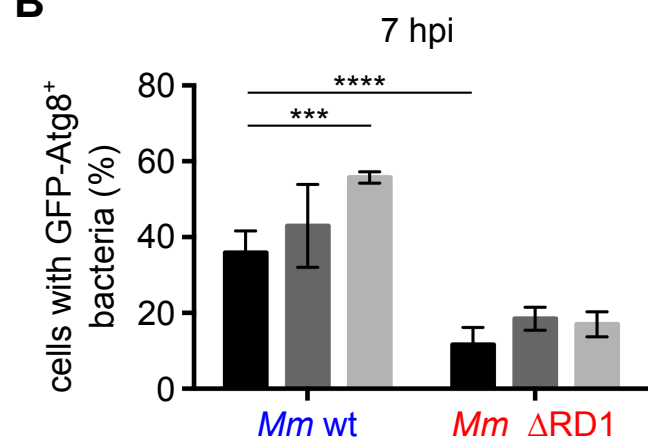

**C**

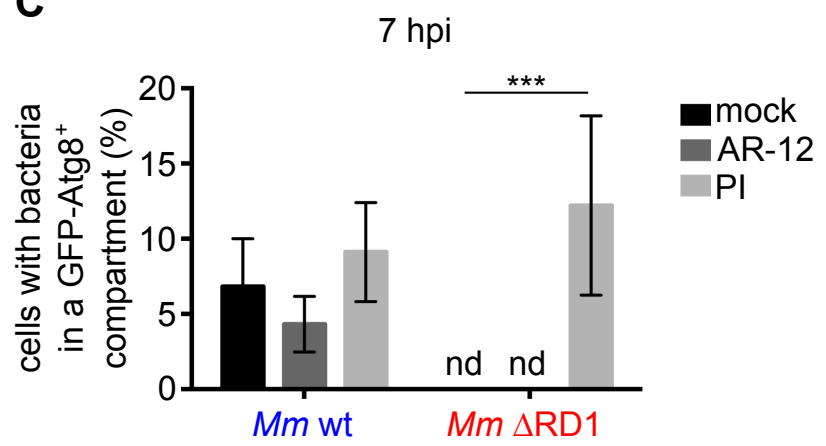

Supplement: S8 Fig — A. EM of D. discoideum cells infected with M. marinum wt for 6 h and treated or not with AR-12 at 2.5 μm for two additional hours. One hour before the end of this treatment, cells were incubated or not with PI at 2.5×. White, green, red and yellow asterisks label bacteria, phagophores and autophagosomes, pycnosomes, and pycnosomes inside autophagosomes, respectively. White arrowheads point to omegasomes-like membrane extensions; green arrowheads indicate sites of double membranes within the MCV. Nuclei are labelled by the letter . Scale bars, 1 μm; B and C. GFP-Atg8-expressing D. discoideum cells were infected for 5 or 6 h with mCherry-expressing M. marinum wt or DsRed-expressing M. marinum ΔRD1 and treated or not with AR-12 at 2.5 μM or PI at 2.5× for 2 or 1 additional hours, respectively. Total time was always 7 hpi. Mean and standard deviation from 2–6 independent replicates of the percentage of cells containing GFP-Atg8+ bacteria (B.) or the percentage of cells containing bacteria enclosed by a GFP-Atg8+ vacuole (C.). A minimum of 163 infected cells was counted per condition in B., while 30–258 cells with GFP-Atg8+ bacteria were counted in C. Unpaired t test (***p ≤ 0.001; ****p ≤ 0.0001). nd: not detected. (PDF) [file ppat.1006344.s009.pdf]

S9 Fig

A

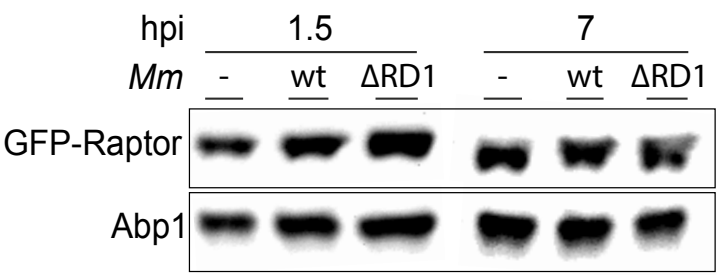

B

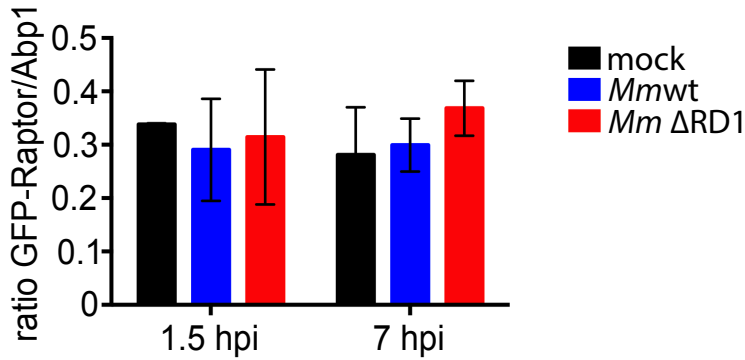

Supplement: S9 Fig — A. GFP-Raptor-expressing D. discoideum cells were infected or not with mCherry-expressing M. marinum wt or with DsRed-expressing M. marinum ΔRD1 for 1.5 and 7 hpi. Representative immunoblots against GFP and Abp1 (loading control) from three independent experiments; B. Mean and standard deviation of the ratio GFP-Raptor/Abp1 from the immunoblots represented in A. (PDF) [file ppat.1006344.s010.pdf]
