# Supplementary material for: Mycobacterium marinum antagonistically induces an autophagic response while repressing the autophagic flux in a TORC1- and ESX-1-dependent manner
Source: PLoS Pathog. 2017 Apr 17;13(4):e1006344. doi: 10.1371/journal.ppat.1006344 (PMC5407849; doi:10.1371/journal.ppat.1006344)
Supplement: S1 Table — (DOCX) [file ppat.1006344.s011.docx]

| **Strain/Plasmid** | **Relevant characteristics** | **Source/Reference** |
| --- | --- | --- |
| *D. discoideum* |  |  |
| Ax2(Ka) | Wild type |  |
| DH1 | Wild type |  |
| Ax2(Ka) *atg1* knock out strain |  | [1] |
| DH1 *atg1* knock out strain |  | Dicty stock center |
| DH1 *atg8* knock out strain |  | Dicty stock center |
| DH1 *p62* knock out strain |  | This study |
| *M. marinum* |  |  |
| M strain | Wild type | L. Ramakrishnan (Washington University) |
| ∆RD1 |  | L. Ramakrishnan (Washington University) |
| ∆CE |  | L.Y. Gao (University of Kentucky) |
| *D. discoideum* plasmids |  |  |
| pDM1045 | Hyg^r^ | [2] |
| pDM1043 | Hyg^r^ | [2] |
| pDM451 | Hyg^r^ | [2] |
| pDXA-GFP | G418^r^ | [3] |
| pJSK500 | GFP-Atg8a | [4] |
| pJSK489 | GFP-Atg18 | [4] |
| pJSK410 | GFP-Ub | This study |
| GFP-p62 | *sqstm1/p62* gDNA (DDB_G0270098) in pDXA-GFP | This study |
| Atg1-GFP |  | Dicty stock center |
| GFP-Rab7a | *rab7A* (DDB_G0269236) in pDXA-GFP | [5] |
| GFP-Rab11c | *rab11C* (DDB_G0277101) in pDXA-GFP | A. Guého |
| VatB-mRFPmars | *vatB* (DDB_G0277401) in pDM451 | [6] |
| Lamtor1-GFP | *lamtor1* gDNA (DDB_G0292160) in pDM1045 | This study |
| GFP-Rheb | *rheb* gDNA (DDB_G0277041) in pDM1043 | This study |
| GFP-Lst8 | *lst8* gDNA (DDB_G0292592) in pDM1043 | This study |
| GFP-Raptor | *raptor* gDNA (DDB_G0270398) in pDM1043 | This study |
| Mycobacteria plasmids |  |  |
| pCherry10 | mCherry under control of the G13 promoter, Hyg^r^ | [7] |
| pMSP12::DsRed/GFP | DsRed/GFP under control of the MSP promoter, Kan^r^ | [8] |
| pGFPHYG2 | Hyg^r^ derivative of pMSP12::GFP | [9] |
| pMV306**::***lux* | bacterial luciferase under control of the G13 promoter, Kan^r^ | [10] |
| pRD1-2F9 | *M. tuberculosis* extended RD1 locus, Amp^r^, Hyg^r^ | [11] |
