# Supplementary material for: Mycobacterium marinum antagonistically induces an autophagic response while repressing the autophagic flux in a TORC1- and ESX-1-dependent manner
Source: PLoS Pathog. 2017 Apr 17;13(4):e1006344. doi: 10.1371/journal.ppat.1006344 (PMC5407849; doi:10.1371/journal.ppat.1006344)
Supplement: S2 Table — (DOCX) [file ppat.1006344.s012.docx]

| **Oligonucleotide/Use** | **Sequence 5'-3' (restriction sites are underlined)** |
| --- | --- |
| qPCR |  |
| gapdhF1 | GGTTGTCCCAATTGGTATTAATGG |
| gapdhR1 | CCGTGGGTTGAATCATATTTGAAC |
| atg1F2 | TCACGCCTCTTCACTTCCTT |
| atg1R2 | CGGTTGTTGGATTGTAGTTGAT |
| atg8aF3 | CTCCAAGATCAGATGCACCA |
| atg8aR3 | GCAGCAGTTGGTGGGATAGT |
| atg8bF4 | GAAATTCCTTGCACCATCAAA |
| atg8bR4 | CGGAACCAAATGTATTTTCACC |
| p62F5 | TTGAAAATCGCACAACCAAC |
| p62R5 | AGGAACCCTTTGGAATGACA |
| Construction of pJSK410 |  |
| ubBamHIfor | GGATCCAAAATGCAAATTTTTGTTAAAACACTTAC |
| ubXbaIrev | TCTAGATTAACCACCTCTTAATCTAAGTAC |
| Construction of GFP-p62 |  |
| p62F10 | CCACTCGAGTAAATCTTATTTTAAAGATCC |
| p62R10 | GACTCTAGATTATTGTTCTTGATTACTTAATAAATGG |
| Construction of Lamtor1-GFP |  |
| lamtor1BglIIfor | GAagatctaaaATGGGTTGCGTTGTTTCAAAATC |
| lamtor1SpeIrev | GGactagtTTTTAAAGAATTTCCAAAAAATACAAC |
| Construction of GFP-Rheb |  |
| rhebBamHIfor | CGggatccaaaATGGCACCACAAAAACATAG |
| rhebSTOPSpeIrev | GGactagtTTACATTAAAATACAACCTTCTTTTTGTG |
| Construction of GFP-Lst8 |  |
| lst8BglIIfor | GAagatctaaaATGCCAGGTATTATATTGGC |
| lst8SpeIrev | GGactagtTCTTGGTAAATCATTTAAAGCAAC |
| Construction of GFP-Raptor |  |
| raptorSpeIfor | GGactagtaaaATGGATAAATCATATAATTTCAGTAG |
| raptorSpeIrev | GGactagtCCAATCAACATTTGATTTTG |
