# Supplementary material for: Mycobacterium marinum antagonistically induces an autophagic response while repressing the autophagic flux in a TORC1- and ESX-1-dependent manner
Source: PLoS Pathog. 2017 Apr 17;13(4):e1006344. doi: 10.1371/journal.ppat.1006344 (PMC5407849; doi:10.1371/journal.ppat.1006344)
Supplement: S3 Table — (DOCX) [file ppat.1006344.s013.docx]

| **Autophagic flux assay** | **λ** |
| --- | --- |
| Fig 6B |  |
| control mock, 1.5 hpi | 1.59 |
| control PI, 1.5 hpi | 4.35 |
| *Mm* wt mock, 1.5 hpi | 3.56 |
| *Mm* wt PI, 1.5 hpi | 5.70 |
| *Mm* ∆RD1 mock, 1.5 hpi | 1.58 |
| *Mm* ∆RD1 PI, 1.5 hpi | 4.53 |
| control mock, 7 hpi | 1.45 |
| control PI, 7 hpi | 3.70 |
| *Mm* wt mock, 7 hpi | 2.78 |
| *Mm* wt PI, 7 hpi | 4.54 |
| *Mm* ∆RD1 mock, 7 hpi | 1.76 |
| *Mm* ∆RD1 PI, 7 hpi | 3.62 |
| S6F Fig |  |
| control mock, wt | 1.67 |
| control PI, wt | 2.62 |
| AR-12 mock, wt | 2.66 |
| AR-12 PI, wt | 4.80 |
| control mock, *atg1*- | 1.91 |
| control PI, *atg1*- | 1.87 |
| AR-12 mock, *atg1*- | 1.80 |
| AR-12 PI, *atg1*- | 2.20 |
| S7C Fig |  |
| wt mock, 1 h | 2.88 |
| wt CMB, 1 h | 3.25 |
| *atg1*- mock, 1 h | 1.58 |
| *atg1*- CMB, 1 h | 1.72 |
| wt mock, 2 h | 2.31 |
| wt CMB, 2 h | 3.59 |
| *atg1*- mock, 2 h | 1.41 |
| *atg1*- CMB, 2 h | 1.70 |
| S7D Fig |  |
| control mock, 2 hpi | 1.84 |
| control CMB, 2 hpi | 3.20 |
| *Mm* wt mock, 2 hpi | 4.60 |
| *Mm* wt CMB, 2 hpi | 5.33 |
| *Mm* ∆RD1 mock, 2 hpi | 2.02 |
| *Mm* ∆RD1 CMB, 2 hpi | 3.34 |
| control mock, 7 hpi | 2.46 |
| control CMB, 7 hpi | 3.60 |
| *Mm* wt mock, 7 hpi | 3.70 |
| *Mm* wt CMB, 7 hpi | 4.38 |
| *Mm* ∆RD1 mock, 7 hpi | 2.01 |
| *Mm* ∆RD1 CMB, 7 hpi | 3.48 |
