# Supplementary material for: Mycobacterium marinum antagonistically induces an autophagic response while repressing the autophagic flux in a TORC1- and ESX-1-dependent manner
Source: PLoS Pathog. 2017 Apr 17;13(4):e1006344. doi: 10.1371/journal.ppat.1006344 (PMC5407849; doi:10.1371/journal.ppat.1006344)
Supplement: S4 Table — (DOCX) [file ppat.1006344.s014.docx]

| **Autophagic flux assay** | **λ_2_ - λ_1_** | ***p*-value** |
| --- | --- | --- |
| Fig 6B |  |  |
|  | (control mock, 1.5 hpi) - (control PI, 1.5 hpi) | 3.52E-08 |
|  | (control mock, 1.5 hpi) - (*Mm* wt mock, 1.5 hpi) | 2.30E-06 |
|  | (control mock, 1.5 hpi) - (*Mm* wt PI, 1.5 hpi) | 3.99E-08 |
|  | (control mock, 1.5 hpi) - (*Mm* ∆RD1 mock, 1.5 hpi) | 9.60E-01 |
|  | (control mock, 1.5 hpi) - (*Mm* ∆RD1 PI, 1.5 hpi) | 3.57E-08 |
|  | (control PI, 1.5 hpi) - (*Mm* wt mock, 1.5 hpi) | 1.11E-03 |
|  | (control PI, 1.5 hpi) - (*Mm* wt PI, 1.5 hpi) | 1.48E-06 |
|  | (control PI, 1.5 hpi) - (*Mm* ∆RD1 mock, 1.5 hpi) | 3.27E-08 |
|  | (control PI, 1.5 hpi) - (*Mm* ∆RD1 PI, 1.5 hpi) | 4.63E-01 |
|  | (*Mm* wt mock, 1.5 hpi) - (*Mm* wt PI, 1.5 hpi) | 1.45E-06 |
|  | (*Mm* wt mock, 1.5 hpi) - (*Mm* ∆RD1 mock, 1.5 hpi) | 2.07E-06 |
|  | (*Mm* wt mock, 1.5 hpi) - (*Mm* ∆RD1 PI, 1.5 hpi) | 2.06E-04 |
|  | (*Mm* wt PI, 1.5 hpi) - (*Mm* ∆RD1 mock, 1.5 hpi) | 3.76E-08 |
|  | (*Mm* wt PI, 1.5 hpi) - (*Mm* ∆RD1 PI, 1.5 hpi) | 5.17E-05 |
|  | (*Mm* ∆RD1 mock, 1.5 hpi) - (*Mm* ∆RD1 PI, 1.5 hpi) | 3.32E-08 |
|  | (control mock, 7 hpi) - (control PI, 7 hpi) | 3.05E-08 |
|  | (control mock, 7 hpi) - (*Mm* wt mock, 7 hpi) | 1.65E-06 |
|  | (control mock, 7 hpi) - (*Mm* wt PI, 7 hpi) | 3.27E-08 |
|  | (control mock, 7 hpi) - (*Mm* ∆RD1 mock, 7 hpi) | 0.044114 |
|  | (control mock, 7 hpi) - (*Mm* ∆RD1 PI, 7 hpi) | 2.74E-08 |
|  | (control PI, 7 hpi) - (*Mm* wt mock, 7 hpi) | 1.33E-05 |
|  | (control PI, 7 hpi) - (*Mm* wt PI, 7 hpi) | 0.001813 |
|  | (control PI, 7 hpi) - (*Mm* ∆RD1 mock, 7 hpi) | 1.75E-06 |
|  | (control PI, 7 hpi) - (*Mm* ∆RD1 PI, 7 hpi) | 0.758755 |
|  | (*Mm* wt mock, 7 hpi) - (*Mm* wt PI, 7 hpi) | 1.05E-06 |
|  | (*Mm* wt mock, 7 hpi) - (*Mm* ∆RD1 mock, 7 hpi) | 1.71E-06 |
|  | (*Mm* wt mock, 7 hpi) - (*Mm* ∆RD1 PI, 7 hpi) | 0.000657 |
|  | (*Mm* wt PI, 7 hpi) - (*Mm* ∆RD1 mock, 7 hpi) | 2.21E-06 |
|  | (*Mm* wt PI, 7 hpi) - (*Mm* ∆RD1 PI, 7 hpi) | 0.003901 |
|  | (*Mm* ∆RD1 mock, 7 hpi) - (*Mm* ∆RD1 PI, 7 hpi) | 1.37E-06 |
| S6F Fig |  |  |
|  | (control mock, wt) - (control PI, wt) | 8.75E-06 |
|  | (control mock, wt) - (AR-12 mock, wt) | 9.26E-06 |
|  | (control mock, wt) - (AR-12 PI, wt) | 2.70E-08 |
|  | (control mock, wt) - (control mock, *atg1*-) | 0.172963 |
|  | (control mock, wt) - (control PI, *atg1*-) | 0.227829 |
|  | (control mock, wt) - (AR-12 mock, *atg1*-) | 0.478464 |
|  | (control mock, wt) - (AR-12 PI, *atg1*-) | 0.004506 |
|  | (control PI, wt) - (AR-12 mock, wt) | 0.870309 |
|  | (control PI, wt) - (AR-12 PI, wt) | 1.26E-06 |
|  | (control PI, wt) - (control mock, *atg1*-) | 0.000262 |
|  | (control PI, wt) - (control PI, *atg1*-) | 5.01E-05 |
|  | (control PI, wt) - (AR-12 mock, *atg1*-) | 7.16E-06 |
|  | (control PI, wt) - (AR-12 PI, *atg1*-) | 0.055026 |
|  | (AR-12 mock, wt) - (AR-12 PI, wt) | 9.31E-07 |
|  | (AR-12 mock, wt) - (control mock, *atg1*-) | 0.000116 |
|  | (AR-12 mock, wt) - (control PI, *atg1*-) | 1.47E-05 |
|  | (AR-12 mock, wt) - (AR-12 mock, *atg1*-) | 4.92E-06 |
|  | (AR-12 mock, wt) - (AR-12 PI, *atg1*-) | 0.032316 |
|  | (AR-12 PI, wt) - (control mock, *atg1*-) | 2.71E-08 |
|  | (AR-12 PI, wt) - (control PI, *atg1*-) | 2.77E-08 |
|  | (AR-12 PI, wt) - (AR-12 mock, *atg1*-) | 2.80E-08 |
|  | (AR-12 PI, wt) - (AR-12 PI, *atg1*-) | 1.88E-06 |
|  | (control mock, *atg1*-) - (control PI, *atg1*-) | 0.751384 |
|  | (control mock, *atg1*-) - (AR-12 mock, *atg1*-) | 0.371510 |
|  | (control mock, *atg1*-) - (AR-12 PI, *atg1*-) | 0.053781 |
|  | (control PI, *atg1*-) - (AR-12 mock, *atg1*-) | 0.536052 |
|  | (control PI, *atg1*-) - (AR-12 PI, *atg1*-) | 0.013065 |
|  | (AR-12 mock, *atg1*-) - (AR-12 PI, *atg1*-) | 0.00372 |
| S7C Fig |  |  |
|  | (wt mock, 1 h) - (wt CMB, 1 h) | 0.033094 |
|  | (wt mock, 1 h) - (*atg1-* mock, 1 h) | 2.02E-06 |
|  | (wt mock, 1 h) - (*atg1-* CMB, 1 h) | 1.48E-06 |
|  | (wt mock, 1 h) - (wt mock, 2 h) | 0.001201 |
|  | (wt mock, 1 h) - (wt CMB, 2 h) | 7.55E-05 |
|  | (wt mock, 1 h) - (*atg1-* mock, 2 h) | 2.72E-08 |
|  | (wt mock, 1 h) - (*atg1-* CMB, 2 h) | 1.18E-06 |
|  | (wt CMB, 1 h) - (*atg1-* mock, 1 h) | 2.90E-08 |
|  | (wt CMB, 1 h) - (*atg1-* CMB, 1 h) | 2.34E-06 |
|  | (wt CMB, 1 h) - (wt mock, 2 h) | 6.24E-07 |
|  | (wt CMB, 1 h) - (wt CMB, 2 h) | 0.048914 |
|  | (wt CMB, 1 h) - (*atg1-* mock, 2 h) | 3.28E-08 |
|  | (wt CMB, 1 h) - (*atg1-* CMB, 2 h) | 2.18E-06 |
|  | (*atg1-* mock, 1 h) - (*atg1-* CMB, 1 h) | 0.074241 |
|  | (*atg1-* mock, 1 h) - (wt mock, 2 h) | 4.91E-07 |
|  | (*atg1-* mock, 1 h) - (wt CMB, 2 h) | 3.24E-08 |
|  | (*atg1-* mock, 1 h) - (*atg1*- mock, 2 h) | 0.011535 |
|  | (*atg1-* mock, 1 h) - (*atg1*- CMB, 2 h) | 0.174880 |
|  | (*atg1-* CMB, 1 h) - (wt mock, 2 h) | 1.11E-05 |
|  | (*atg1-* CMB, 1 h) - (wt CMB, 2 h) | 3.09E-08 |
|  | (*atg1-* CMB, 1 h) - (*atg1*- mock, 2 h) | 7.62E-06 |
|  | (*atg1-* CMB, 1 h) - (*atg1*- CMB, 2 h) | 0.811128 |
|  | (wt mock, 2 h) - (wt CMB, 2 h) | 1.36E-06 |
|  | (wt mock, 2 h) - (*atg1*- mock, 2 h) | 1.19E-06 |
|  | (wt mock, 2 h) - (*atg1*- CMB, 2 h) | 6.80E-05 |
|  | (wt CMB, 2 h) - (*atg1*- mock, 2 h) | 3.60E-08 |
|  | (wt CMB, 2 h) - (*atg1*- CMB, 2 h) | 2.81E-08 |
|  | (*atg1*- mock, 2 h) - (*atg1*- CMB, 2 h) | 0.000326 |
| S7D Fig |  |  |
|  | (control mock, 1.5 hpi) - (control CMB, 1.5 hpi) | 2.06E-06 |
|  | (control mock, 1.5 hpi) - (*Mm* wt mock, 1.5 hpi) | 3.24E-08 |
|  | (control mock, 1.5 hpi) - (*Mm* wt CMB, 1.5 hpi) | 3.53E-08 |
|  | (control mock, 1.5 hpi) - (*Mm* ∆RD1 mock, 1.5 hpi) | 0.161717 |
|  | (control mock, 1.5 hpi) - (*Mm* ∆RD1 CMB, 1.5 hpi) | 1.93E-06 |
|  | (control CMB, 1.5 hpi) - (*Mm* wt mock, 1.5 hpi) | 1.08E-06 |
|  | (control CMB, 1.5 hpi) - (*Mm* wt CMB, 1.5 hpi) | 2.19E-06 |
|  | (control CMB, 1.5 hpi) - (*Mm* ∆RD1 mock, 1.5 hpi) | 1.22E-06 |
|  | (control CMB, 1.5 hpi) - (*Mm* ∆RD1 CMB, 1.5 hpi) | 0.456937 |
|  | (*Mm* wt mock, 1.5 hpi) - (*Mm* wt CMB, 1.5 hpi) | 0.013157 |
|  | (*Mm* wt mock, 1.5 hpi) - (*Mm* ∆RD1 mock, 1.5 hpi) | 2.85E-08 |
|  | (*Mm* wt mock, 1.5 hpi) - (*Mm* ∆RD1 CMB, 1.5 hpi) | 2.11E-06 |
|  | (*Mm* wt CMB, 1.5 hpi) - (*Mm* ∆RD1 mock, 1.5 hpi) | 3.18E-08 |
|  | (*Mm* wt CMB, 1.5 hpi) - (*Mm* ∆RD1 CMB, 1.5 hpi) | 1.11E-06 |
|  | (*Mm* ∆RD1 mock, 1.5 hpi) - (*Mm* ∆RD1 CMB, 1.5 hpi) | 1.15E-06 |
|  | (control mock, 7 hpi) - (control CMB, 7 hpi) | 1.29E-06 |
|  | (control mock, 7 hpi) - (*Mm* wt mock, 7 hpi) | 1.08E-06 |
|  | (control mock, 7 hpi) - (*Mm* wt CMB, 7 hpi) | 2.31E-06 |
|  | (control mock, 7 hpi) - (*Mm* ∆RD1 mock, 7 hpi) | 0.019765 |
|  | (control mock, 7 hpi) - (*Mm* ∆RD1 CMB, 7 hpi) | 5.50E-07 |
|  | (control CMB, 7 hpi) - (*Mm* wt mock, 7 hpi) | 0.618045 |
|  | (control CMB, 7 hpi) - (*Mm* wt CMB, 7 hpi) | 0.000423 |
|  | (control CMB, 7 hpi) - (*Mm* ∆RD1 mock, 7 hpi) | 9.92E-07 |
|  | (control CMB, 7 hpi) - (*Mm* ∆RD1 CMB, 7 hpi) | 0.600902 |
|  | (*Mm* wt mock, 7 hpi) - (*Mm* wt CMB, 7 hpi) | 0.008557 |
|  | (*Mm* wt mock, 7 hpi) - (*Mm* ∆RD1 mock, 7 hpi) | 8.39E-07 |
|  | (*Mm* wt mock, 7 hpi) - (*Mm* ∆RD1 CMB, 7 hpi) | 0.377875 |
|  | (*Mm* wt CMB, 7 hpi) - (*Mm* ∆RD1 mock, 7 hpi) | 1.62E-06 |
|  | (*Mm* wt CMB, 7 hpi) - (*Mm* ∆RD1 CMB, 7 hpi) | 0.000827 |
|  | (*Mm* ∆RD1 mock, 7 hpi) - (*Mm* ∆RD1 CMB, 7 hpi) | 6.50E-07 |
